# Age modifies respiratory complex I and protein homeostasis in a muscle type‐specific manner

**DOI:** 10.1111/acel.12412

**Published:** 2015-10-25

**Authors:** Shane E. Kruse, Pabalu P. Karunadharma, Nathan Basisty, Richard Johnson, Richard P. Beyer, Michael J. MacCoss, Peter S. Rabinovitch, David J. Marcinek

**Affiliations:** ^1^ Department of RadiologyUniversity of WashingtonSeattleWAUSA; ^2^ Department of PathologyUniversity of WashingtonSeattleWAUSA; ^3^ Scripps Research InstituteJupiterFLUSA; ^4^ Department of Genome SciencesUniversity of WashingtonSeattleWAUSA; ^5^ Department of Environmental and Occupational Health SciencesUniversity of WashingtonSeattleWAUSA; ^6^ Department of BioengineeringUniversity of WashingtonSeattleWAUSA

**Keywords:** aging, mitochondria, mitochondrial dysfunction, protein turnover, proteome, skeletal muscle

## Abstract

Changes in mitochondrial function with age vary between different muscle types, and mechanisms underlying this variation remain poorly defined. We examined whether the rate of mitochondrial protein turnover contributes to this variation. Using heavy label proteomics, we measured mitochondrial protein turnover and abundance in slow‐twitch soleus (SOL) and fast‐twitch extensor digitorum longus (EDL) from young and aged mice. We found that mitochondrial proteins were longer lived in EDL than SOL at both ages. Proteomic analyses revealed that age‐induced changes in protein abundance differed between EDL and SOL with the largest change being increased mitochondrial respiratory protein content in EDL. To determine how altered mitochondrial proteomics affect function, we measured respiratory capacity in permeabilized SOL and EDL. The increased mitochondrial protein content in aged EDL resulted in reduced complex I respiratory efficiency in addition to increased complex I‐derived H_2_O_2_ production. In contrast, SOL maintained mitochondrial quality, but demonstrated reduced respiratory capacity with age. Thus, the decline in mitochondrial quality with age in EDL was associated with slower protein turnover throughout life that may contribute to the greater decline in mitochondrial dysfunction in this muscle. Furthermore, mitochondrial‐targeted catalase protected respiratory function with age suggesting a causal role of oxidative stress. Our data clearly indicate divergent effects of age between different skeletal muscles on mitochondrial protein homeostasis and function with the greatest differences related to complex I. These results show the importance of tissue‐specific changes in the interaction between dysregulation of respiratory protein expression, oxidative stress, and mitochondrial function with age.

## Introduction

Age‐induced changes in skeletal muscle have significant public health consequences due to increased frailty and weakness leading to loss of independence and increased risk of falls (Faulkner *et al*., 2007). This is in addition to the comorbidities associated with the lack of physical activity. Almost 50 million Americans are projected to be elderly (over 65 years of age) in 2015, and the elderly age bracket is increasing at a rate 56% faster than all other age groups (US Census Bureau). This age group also has a higher incidence of disease and injury and as much as 10 million of the elderly population is classified as frail (Bortz, 2002). The annual economic burden of sarcopenia, a correlate of musculoskeletal aging and frailty, had already reached 18 billion dollars in 2001 (Janssen *et al*., 2006).

Skeletal muscle relies on mitochondria to meet the majority of the high ATP demand generated during sustained contractile activity associated with many activities of daily living. Therefore, muscle mitochondrial content and function are directly linked to muscle performance, metabolic disease risk, and quality of life (Conley *et al*., 2000; Short *et al*., 2005). Both *in vivo* and ex vivo approaches indicate that skeletal muscle mitochondrial function declines with age in both rodents and humans (Conley *et al*., 2000; Amara *et al*., 2007; Siegel *et al*., 2012; Gouspillou *et al*., 2014). In addition to their role as the primary site of ATP production in most cells, mitochondria are also critical for cell health and function due to their role in controlling apoptosis, calcium buffering, redox signaling, differentiation, and growth. Thus, mitochondria are intimately synchronized with and influence cellular protein homeostasis, not only because of their unique position as sensors of environmental change, but also because over 99% of mitochondrial proteins are nuclear in origin. A recent proteomic analyses of aging heart and liver using whole tissue homogenates indicated that mitochondrial‐related proteins were the most prominent groups affected by age in both abundance and half‐life (Dai *et al*., 2014; Karunadharma *et al*., 2015).

A responsive proteome is essential to meet the changing demands of muscle growth, repair, and exercise. Protein homeostasis is the result of the balance between of protein synthesis, processing, transportation, and degradation. Diminished protein homeostasis with age is implicated in the decline of skeletal muscle function and atrophy. Aging negatively impacts the expression of many genes in skeletal muscle that are involved in protein homeostasis (Johnson *et al*., 2014), and defects in protein degradation cause myopathy (Lin *et al*., 2015). Conversely, interventions such as exercise and dietary restriction that improve aged skeletal muscle function also restore protein homeostasis (Kumar *et al*., 2009; Garvey *et al*., 2015). Mitochondrial dysfunction in particular is associated with a decline in protein quality control and autophagy in aging tissues (Schleit *et al*., 2013; Dai *et al*., 2014). However, the relationship between mitochondrial protein turnover, accumulation, and tissue‐specific variation in mitochondrial dysfunction remains poorly described.

The effect of age on mitochondrial content and function varies with muscle type and the physiological demands of the muscle (Holloszy, 1967; Picard *et al*., 2012). Skeletal muscle can be classified into two broad categories; type I or slow twitch and type II or fast twitch. Type II muscle can be further categorized into type IIA fast oxidative muscle (or intermediate fast twitch) that is fatigue resistant similar to type I, and the fatigable type IIB and IIX fast glycolytic fibers. The response to injury and atrophy varies with muscle fiber type. Type I muscle fibers may be more susceptible to atrophy from denervation and inactivity or microgravity (Grossman *et al*., 1998; Patterson *et al*., 2006), while type II fast‐twitch muscle fibers preferentially atrophy with age, sepsis, nutrient deprivation, and diabetes (Verdijk *et al*., 2007; Jerkovic *et al*., 2009; Picard *et al*., 2011). Although the literature suggests that age‐related mitochondrial dysfunction is greater in fast‐twitch muscles, a mechanistic explanation of how the distinct composition and metabolic activity of muscle fiber type drive the specific sensitivity to various physiological insults has not been well defined.

In this study, we use the extensor digitorum longus (EDL) and soleus (SOL) muscles in a mouse model of aging to represent the range of muscle phenotypes. The EDL is primarily a fast‐twitch muscle with lower mitochondrial content (0.4% type I, 0.5% IIA, and the remainder mostly type IIB or other ‘fast‐twitch’ type IIB/X), while the SOL is approximately 40% type I and 40% IIA with few IIB/X fibers and a higher mitochondrial content (Augusto, Padovani *et al*. 2004). This divergence of muscle fiber type thus represents a good model for testing the interaction between aging and muscle type. Due to the important role of mitochondria in integrating energy metabolism and cell signaling through control of cell redox and metabolite levels, as well as ATP supply, it is important to examine changes in both mitochondrial quality and capacity in response to aging and disease. Both parameters will be affected by changes in protein homeostasis, reflected in changes in both mitochondrial protein abundance and turnover. Aging is associated with altered mitochondrial protein homeostasis with documented effects on both biogenesis and mitochondrial autophagy (mitophagy) (Lee *et al*., 2002; Masiero *et al*., 2009; Schneider *et al*., 2014).

Respiratory dysfunction accompanies aging and complex I function is a particularly important component of longevity and health (Kirby *et al*., 1999; Kruse *et al*., 2008; Miwa *et al*., 2014). Altered respiratory activity results in various phenotypes that are dependent on tissue type, penetrance of mitochondrial dysfunction, genetic background, and biochemical defect. This suggests that indirect as well as direct consequences of mitochondrial dysfunction affect the cell and organism. Mitochondrial dysfunction can also reach across point of origin to the entire organism affecting distal tissues. Inhibition of over half the maximal activity of complex I does not necessarily result in a commensurate decrease of *in vivo* basal ATP synthase activity (Kruse *et al*., 2008). However, changes in complex I stoichiometry can affect the efficiency of electron transfer and result in oxidative stress (Miwa *et al*., 2014) and slight alterations of the redox status of electron donors such as NAD/NADH can have genomewide effects (Imai & Guarente, 2014). Mitochondrial dysfunction is repeatedly at the forefront of age‐related disease. Complex I deficiencies alone account for approximately half all defects in oxidative phosphorylation (Kirby *et al*., 2004; McFarland *et al*., 2004) where prognosis is poor and is often lethal.

Despite intense research into the biochemistry of mitochondrial energetics, there are relatively few studies examining mitochondrial protein homeostasis in skeletal muscle. Here, we examine age‐related changes in abundance and turnover in the mitochondrial proteome and relate these changes in protein homeostasis to changes in mitochondrial function in disparate muscle types. We hypothesized that differences in mitochondrial protein homeostasis would underlie muscle‐specific mitochondrial deficits in aging skeletal muscle.

## Results

### Muscle‐specific differences in mitochondrial protein half‐lives

**Figure 1:**
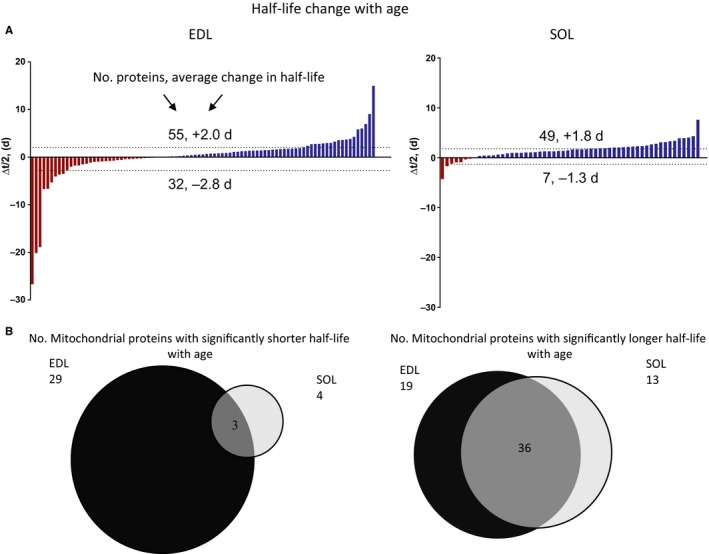
Mitochondrial Protein Half‐life is Modified by Age and is Dependent on Muscle Type. (A) Plots showing significant changes in mitochondrial protein half‐life with age. Changes were roughly half increasing and half decreasing in half‐life in EDL, while the majority of mitochondrial proteins in SOL increased half‐life (slower turnover rate). Proteins with half‐life longer than one year were excluded from the analyses. (B) Venn diagrams showing few mitochondrial proteins decreased half‐life in aged SOL, whereas those that increased half‐life were similar to those that increased half‐life with age in EDL. q value < 0.05.

To assess the effects of age on mitochondrial protein expression, we used a proteomic approach to measure changes in protein abundance and turnover in mitochondria‐enriched fractions from EDL and SOL of young (5–8 months) and aged (27–29 months) female mice. Protein half‐lives were measured by feeding mice with ^2^H_3_‐labeled leucine diet and quantitating the rate of appearance of newly synthesized proteins by mass spectrometry and the software tool Topograph (see methods). Topograph allows the correct quantitation of the percent newly synthesized proteins, even if the precursor pool label abundance differs between groups. By examining the change in newly synthesized proteins over time, protein half‐lives are accurately measured (Hsieh *et al*., 2012). The effect of age on protein half‐lives differed between the EDL and SOL (Fig. 1A and B). Canonical pathway analysis using the IPA library identified the pathways that were significantly changed with age (Fig. S1 and Table S1).

In both muscles, mitochondrial proteins were longer lived than those of the total homogenate (*P *< 0.0001) and also longer lived than cytosolic proteins (*P* < 0.0001) and this persisted with age (Tables 1 and S2). Mean half‐lives did not follow the same trend as the median due to the exceptionally long half‐life (over 100 days) of 18 cytosolic proteins. Mitochondrial proteins were longer lived in the young EDL than in SOL, and this difference also persisted with age. Separating proteins into categories of those that either significantly increased or decreased half‐life with age revealed a much larger effect of age on protein turnover than was reflected in the overall means (Fig. 1). In the SOL, nearly all mitochondrial proteins became longer lived with age (49 longer to 7 shorter lived), while the proteins were more evenly split in the EDL (55 to 32).

**Table 1:** Protein Half‐life^a^

	EDL Proteins median, mean t/2 (d) (No.)	SOL Proteins median, mean t/2 (d) (No.)
Total homogenate, young	22.9, 29.1 (507)	18.0, 23.8 (508)
Total homogenate, aged	23.6, 30.6 (506)	19.2, 25.5 (508)
mito, young	24.9, 25.0 (175)	22.3, 22.3 (182)
mito, aged	25.9, 25.8 (175)	24.1, 24.3 (182)
cyto, young	22.2, 27.7 (107)	16.4, 25.5 (106)
cyto, aged	22.8, 28.6 (107)	16.8, 24.4 (106)

a 745 proteins detected, 15 proteins with half‐life > 1 year removed from analyses

### Mitochondrial protein content changes with age and is divergent between EDL and SOL

#### Mitochondrial protein content is increased in aged EDL

**Figure 2:**
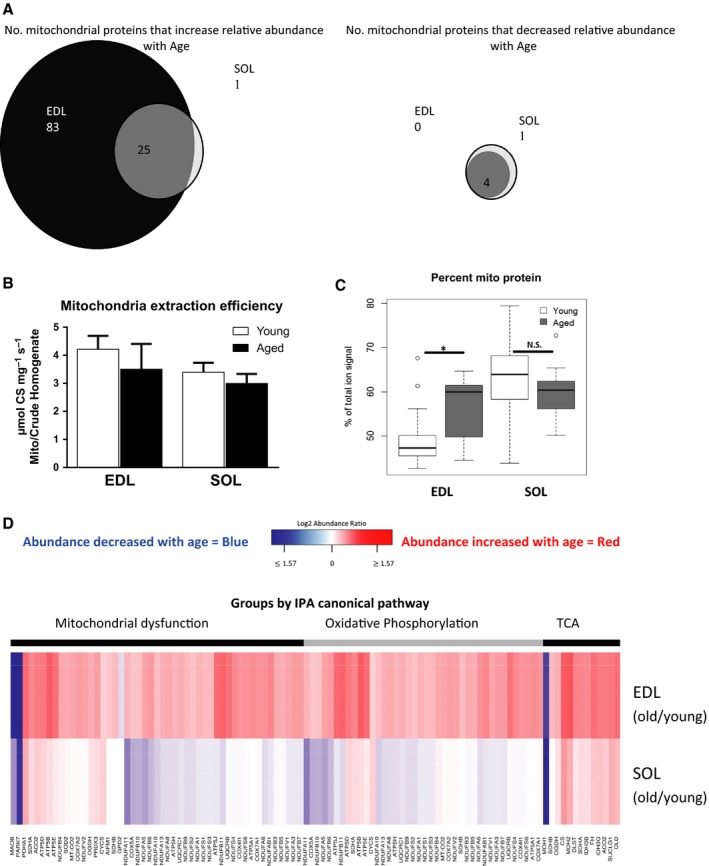
Protein Abundance is Modified by Age and Muscle Type (A) 137 total proteins significantly (adjusted *P* value [q value] < 0.05) increased in content with age in EDL among 745 total proteins detected. 108 of these proteins were mitochondrial, comprising over 96% of mitochondrial proteins that changed with age in the EDL. Black ‐ EDL only, dark grey ‐ EDL and SOL, light grey ‐ SOL only. (B) Comparing citrate synthase activity between mitochondrial enriched fractions and whole cell homogenate shows that the efficiency of extraction was not different in young vs. aged tissues and was not different between EDL and SOL in either age group. (C) Measurement of the AUC of all mitochondrial proteins identified using on an online database (mitoP2, http://www.hsls.pitt.edu/obrc/index.php?page=;URL1097158105) relative to total AUC indicates that mitochondrial content of aged EDL is increased relative to young EDL. * *P* < 0.05. (D) The direction of change with age was often different between EDL and SOL. Heat map of old/young ratio of protein abundance grouped by Ingenuity Pathway Analyses (IPA) canonical pathways. Top three pathways shown, ordered left to right by the significance of the pathway change with age, q < 0.05 increased with age in darker red, and decreased with age in darker blue. Some proteins occur in more than one pathway. EDL top, SOL bottom rows. IPA of Proteins that change abundance with age, q < 0.05, excluding pathways with less than 4 gene products that changed with age are listed in Table S2.

We also observed intermuscle variation in the effect of age on mitochondrial protein abundance. Significant age‐related changes in relative protein abundances occurred in both EDL (28.6% of detected proteins) and SOL (10.9% of detected proteins). Relative expression of mitochondrial proteins increased with age more in the EDL than SOL (Fig. 2A), although both muscles underwent atrophy with age (Fig. S2). To verify that the intermuscle differences in mitochondrial protein expression were not an artifact of our enrichment procedure, we compared citrate synthase activity between homogenates and mitochondrial‐enriched samples for all groups, demonstrating that our efficiency of mitochondrial enrichment was not different between young and aged muscles (Fig. 2B). Comparing the total area under the curve (AUC) for mitochondrial proteins and nonmitochondrial proteins in the MS spectra shows the expected higher AUC for mitochondrial proteins in the young SOL than the young EDL, reflecting the greater mitochondrial content in the slow‐type muscle in the young mice. The AUC data also support an overall increased abundance of mitochondrial proteins with age in the EDL while that of the SOL was unchanged (Fig. 2C). More specifically, proteins involved in oxidative phosphorylation increased in abundance in the EDL, but not the SOL, while TCA proteins were increased with age in both muscles (Fig. 2D). Abundance of mitochondrial chaperones, including prohibitin, HSP60, HSP70, and HSP10, was also increased in aged EDL (Table S2). Changes in protein abundance were largely independent from changes in half‐lives; the correlation (*r* ^2^) between the two was only 2–3% (Fig. S3).

### Respiratory capacity in aged skeletal muscle is preserved in EDL but not SOL

#### Reduced respiratory efficiency with age in EDL: increased respiratory protein content and no change in respiratory flux

**Figure 3:**
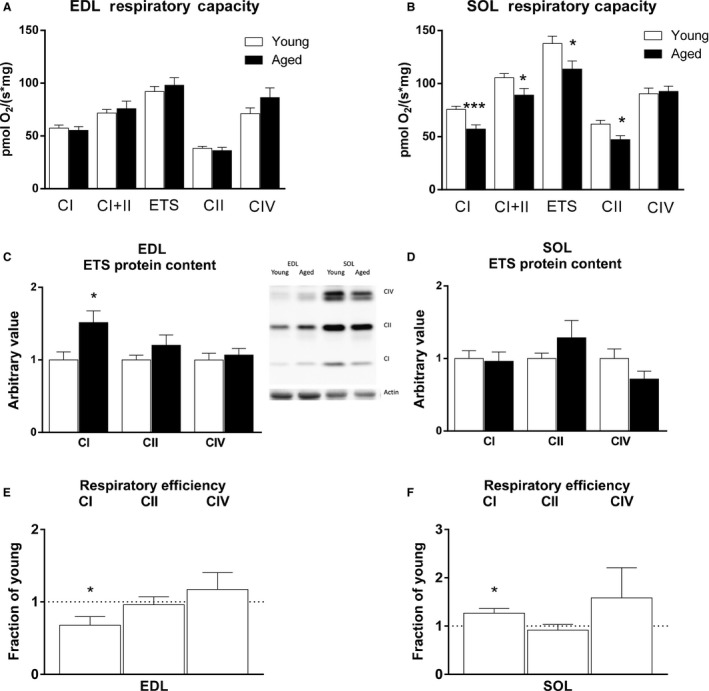
Age Causes Changes in Respiratory Capacity and Mitochondrial Content. Respiration in fast twitch EDL (A) was maintained with age, but decreased in slow twitch SOL (B), *n* = 12─24. Mitochondrial respiratory protein content of representative subunits increased with age in EDL (C) while stable or decreased in SOL (D), *n* = 10─17. Inset shows western analyses of respiratory components CI subunit NDUFB8, CII 30 kDa subunit, and CIV subunit I. 42 kD Ponceau (actin) band was used to normalize protein load. Data are expressed relative to young EDL or SOL. Respiratory flux per mitochondrial content is expressed as a fraction of the young control value (E and F), *n *= 8. * *P *< 0.05, *** *P *< 0.001.

To determine the functional effect of differences in protein homeostasis with age in these two muscles, we measured respiratory capacity in the EDL (low mitochondrial content) and soleus (high mitochondrial content) muscles. Maximal respiration in the presence of complex I substrates (CI and CI+II State 3) for young and aged EDL was not different (Fig. 3A), while respiration declined with age in the mitochondria‐rich soleus (Fig. 3B). Uncoupling oxidation from phosphorylation to measure maximal electron transport (ETS) flux showed the same pattern with maintained flux with age in EDL while ETS flux was decreased with age in SOL. When complex I was bypassed to measure uncoupled complex II, there was also a decrease with age in SOL, and complex IV flux was not different with age in either EDL or SOL. These differences in the effect of age on mitochondrial function between fast‐ and slow‐type skeletal muscles are consistent with previous work in aging rat skeletal muscle (Picard *et al*., 2011).

### Mitochondrial protein content is increased in EDL but not SOL

We confirmed that the effect of age on expression of mitochondrial respiratory proteins was muscle type dependent by Western analyses. A representative subunit of complex I (NdufB8) was significantly elevated with age in the EDL (Fig. 3C). In contrast to the EDL, electron transport proteins in the SOL were stable with age (Fig. 3D) thus confirming the proteomics data. Mitochondrial content alone is not always indicative of mitochondrial health and respiratory capacity on its own does not provide a measure of mitochondrial efficiency when mitochondrial content may be altered. Therefore, age‐induced changes in respiratory capacity were assessed relative to the protein content of that respiratory component. When compared to young levels, complex I respiratory efficiency was reduced with age in the EDL (Fig. 3E), but not in the SOL (Fig. 3F).

### Mitochondrial complex I is selectively altered in aged EDL

**Figure 4:**
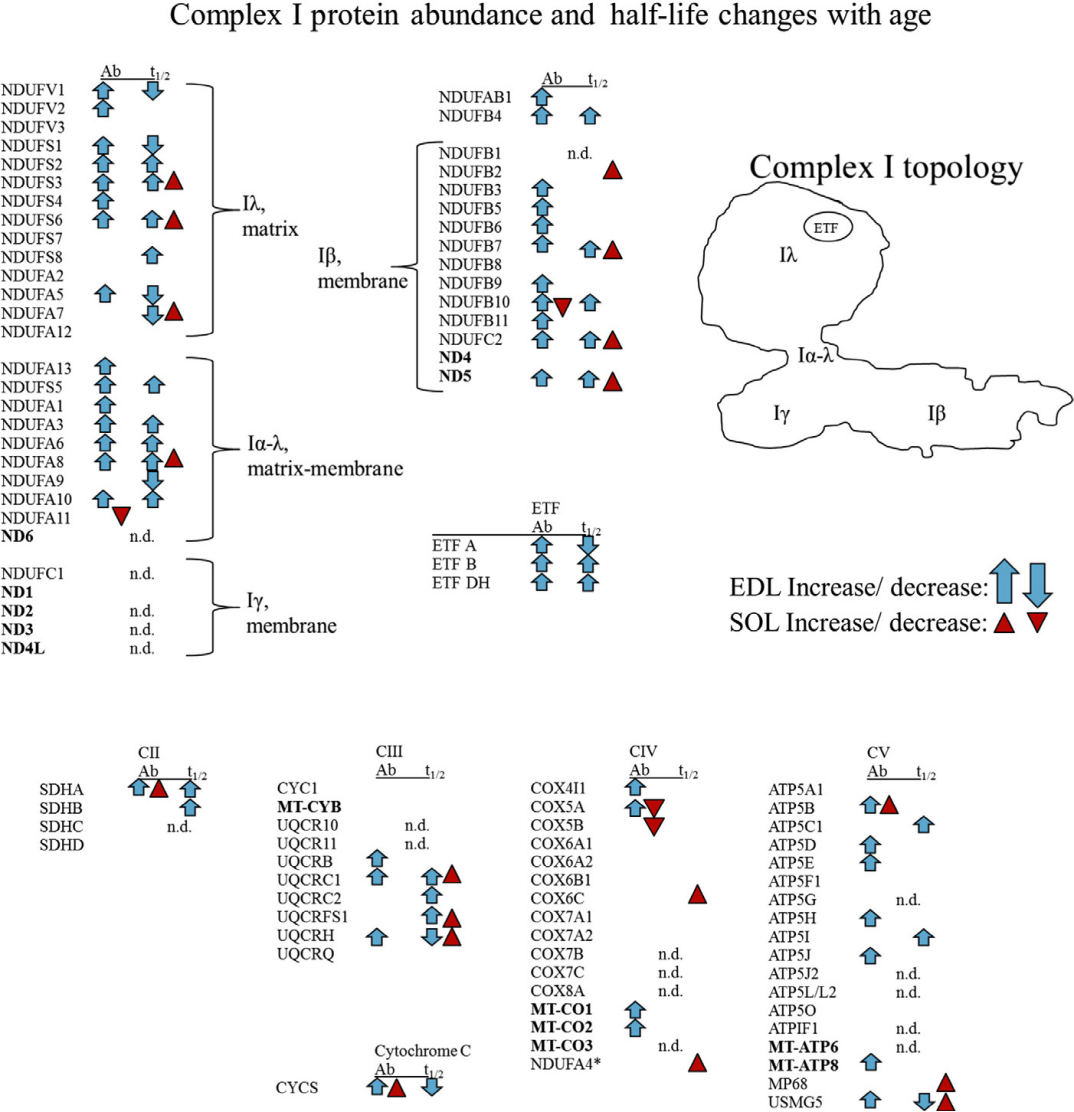
Effect of Age on Expression and Half‐life of ETS Components in the EDL. Map of complex I respiratory apparatus, electron transfer flavin (ETF) moiety and table of other respiratory complexes (mitochondria‐encoded in bold) showing changes in protein abundance (Ab) and half‐life (t_1/2_). *NDUFA4, traditionally associated with complex I, may be associated instead with complex IV (Balsa *et al*. 2012). n.d. = not detected.

The proteomics analyses revealed that many proteins involved in respiration changed with age in either abundance or turnover rate. Therefore, we looked at submitochondrial localization of these proteins. Respiratory complex I contains at least 44 different proteins residing in either the matrix arm or hydrophobic intermembrane region of the holocomplex (Smeitink *et al*., 2001; Janssen *et al*., 2006). Thirty‐eight of the 44 complex I proteins were detected in our proteomic analyses. In aged SOL, the only significant complex I abundance changes were two proteins with decreased content. In contrast, in aged EDL, 29 complex I proteins (including the complex I electron transfer flavoprotein prosthetic group; ETF) were increased in abundance and none were decreased. Only one of these, ND5, is mitochondria encoded. These proteins were distributed throughout complex I in both hydrophilic and intermembrane regions (Fig. 4 and Tables S2 and S3). However, altered protein turnover with age was concentrated to the matrix arm where 14 of 23 matrix arm proteins detected were significantly different with age in the EDL. These included almost every iron–sulfur‐containing protein. Eleven of these 14 proteins also increased in abundance with age. Of the 41 proteins detected in complex I /ETF, 32 (78%) were altered in abundance and/or turnover in aged EDL. Proteins in other respiratory complexes were also different with age in either abundance or turnover although fewer subunits were altered. The complex I‐specific assembly factor ACAD9 was also increased in abundance with age. Consistent with the rest of the mitochondrial proteome, age‐induced changes in abundance of complex I subunits correlated poorly with changes in half‐life (EDL Y = −0.1169*X – 0.3905, *r* ^2^ = 0.06757, *P* = 0.1257; SOL Y = 0.4964*X + 0.1283, *r* ^2^ = 0.05241, *P* = 0.1928), that is increased abundance in complex I subunits of EDL did not predict direction of change in half‐life. Aged SOL displayed consistently increased half‐life with age in 8 proteins of complex I, but these were not specifically localized to the matrix arm.

### Oxidative state suggests greater stress in aged EDL

#### Mitochondrial H_2_O_2_ production and protein modifications

To determine whether altered protein content correlated with increased oxidative stress, we measured H_2_O_2_ production, protein carbonyl content, and protein S‐glutathionylation. Mitochondrial H_2_O_2_ production measured in permeabilized muscle increased with age in both muscles relative to muscle wet weight (Fig. S4A). However, when expressed relative to mitochondrial content (VDAC1 expression), only EDL showed a significant elevation of mitochondrial H_2_O_2_ production with age (Fig. S4B). Oxidative protein damage, as measured by protein carbonylation showed a trend to be elevated in the EDL with age, but no significant differences were observed (Fig. S4C). Protein S‐glutathionylation, a reversible post‐translational modification of protein cysteine residues, increased with age in only SOL (Fig. S4D).

### Mitochondrial‐targeted catalase reverses complex I respiratory dysfunction

**Figure 5:**
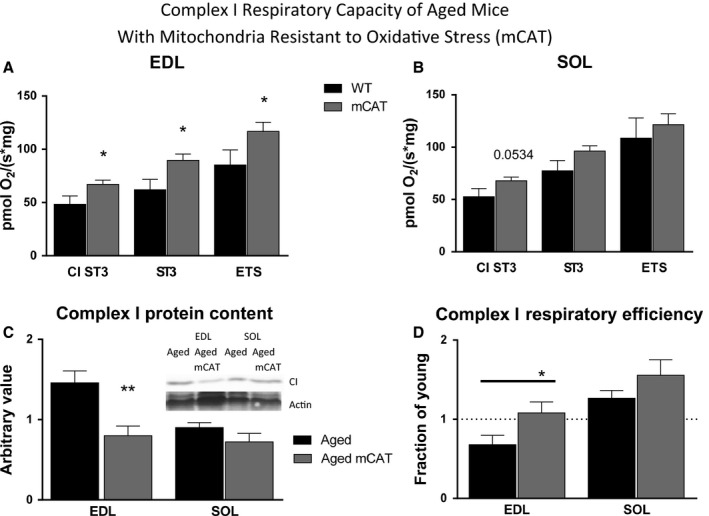
Improved Age Related Dysfunction of Complex I Respiratory Capacity, Efficiency and Protein Content in mCAT Mice. Respiration in aged mCAT mice (A) was elevated in EDL and (B) not significantly changed in SOL compared to WT,* n* = 5─12. (C) Complex I content of aged EDL in mCAT mice is decreased compared to WT aged mice (aged from Fig. 3C). Inset shows western analyses of CI subunit NDUFB8. 42 kD Ponceau (actin) band was used to normalize protein load. Shown relative to young EDL or SOL,* n* = 11. (D) Complex I respiratory flux per mitochondrial content of NdufB8 is expressed as a fraction of the young control value (aged from Fig. 3D). All data sets in figure D were significantly different than hypothetical mean of 1 (young) with the exception of aged mCAT EDL, which was not different than young, but was significantly different from aged WT mice (T‐test), *n* = 8. * *P *< 0.05, ** *P *< 0.01 compared to WT.

To determine whether resistance to oxidative stress could ameliorate age‐induced respiratory dysfunction, we measured respiratory capacity and respiratory complex I protein content in skeletal muscle of adult and aged mice expressing catalase targeted to mitochondria (mCAT mice; Schriner *et al*., 2005). Complex I respiratory dysfunction and protein content were improved in aged mCAT relative to wild‐type EDL (Fig. 5).

## Discussion

Our data demonstrate that the effects of age on protein homeostasis and mitochondrial respiratory function are muscle type specific and highlight the central role of complex I dysfunction. We observed a loss of mitochondrial quality, a compensatory increase in content of mitochondrial proteins that maintained respiratory capacity, and elevated mitochondrial H_2_O_2_ production only in the EDL. Decreased mitochondrial content and functionality are considered to be common pathophysiological effects in aging skeletal muscle. However, we show here that mitochondrial content was increased, in particular for respiratory complex I, and compensated for the reduced respiratory efficiency in the mouse EDL resulting in a lower respiratory capacity per mitochondria (lower respiratory efficiency) without change in total respiratory capacity. In contrast, the SOL demonstrated minimal effect of age on respiratory efficiency and mitochondrial protein expression, while undergoing a loss of complex I‐dependent respiratory capacity. These data suggest that the increased abundance of mitochondrial complex I proteins in aged EDL is an adaptive response to loss of mitochondrial quality, whereas the SOL is either unable to make or requires fewer compensatory changes in mitochondrial protein content with age. In the EDL, the reduced respiratory efficiency with age was associated with a reduced turnover (longer half‐lives) of mitochondrial proteins in adults and aged mice and is thus consistent with a relationship between the effect of age on mitochondrial quality and the rate of mitochondrial protein turnover in skeletal muscle. Our data suggest that slower mitochondrial protein turnover is associated with greater mitochondrial dysfunction and emphasize the important role of respiratory complex I.

Complex I was the most affected protein complex with 78% of subunits changing in either abundance or half‐life with age in the EDL. Complex I is the largest and most intricate respiratory enzyme, the primary entry site of electrons into the respiratory chain, implicated in many disease states, including Leigh syndrome, and the enzyme most often affected in respiratory disorders (Kirby *et al*., 1999; Loeffen *et al*., 2000; Smeitink *et al*., 2001; Kruse *et al*., 2008). Abundance of every complex I subunit in the EDL was increased among those that showed any change in abundance with age. The increased abundance was independent of changes in protein half‐life, which was altered in both directions in many subunits. These data support the hypothesis that complex I subunits are individually replaced rather than recycled as part of a holocomplex (Karunadharma *et al*., 2015). There are 14 core complex I proteins conserved in eukaryotes and necessary for catalysis; 7 are encoded by the mitochondria and 7 by the nucleus. The core proteins encoded in the nucleus include iron–sulfur (Fe‐S)‐containing proteins necessary for electron transfer and are located in the Iλ matrix arm of complex I, such as NDUFV1 which contains the NADH‐binding site as well as flavin mononucleotide (FMN)‐ and Fe‐S‐binding sites (Hinchliffe & Sazanov, 2005) (EC 1.6.5.3, 1.6.99.3). In EDL, 6 of 7 core proteins encoded by the nucleus increased in abundance with age. Furthermore, our data suggest that mitochondrial chaperones critical for folding and assembly of newly imported proteins into the mitochondria such as prohibitin, HSP60, HSP70, and HSP10 are increased in relative abundance in aged EDL. Indeed, the structurally and functionally unique mitochondrial chaperones HSP60 and HSP70, essential components of the mitochondrial unfolded protein response, both stabilize partially unfolded proteins and prevent protein aggregation and degradation (Bukau & Horwich, 1998). Finally, increased relative abundance was observed for ACAD9 a complex I‐specific assembly factor, but not for assembly factors specific to other respiratory complexes. Mitochondrial‐encoded subunit ND‐1 is also essential for complex I assembly. However, neither abundance nor turnover of ND‐1 changed with age in either muscle. The nonuniform effects of age on complex I subunit abundance reported here are consistent with the loss of coordination between nuclear and mitochondrial‐encoded subunits of the respiratory chain recently described in aging muscle (Gomes *et al*., 2013; Miwa *et al*., 2014), although the change reported here (increased nuclear‐encoded and ND5 subunits) was dissimilar to that reported by Gomes *et al*. (decreased mitochondrial‐encoded subunits). Increased content of complex I proteins with age, specifically those of the matrix arm, has been linked to decreased lifespan and oxidative stress (Miwa *et al*., 2014); thus, a breakdown in stoichiometry in respiratory subunits is expected to lead to poor assembly and function of complex I that would result in the decreased mitochondrial respiratory efficiency and elevated mitochondrial oxidative stress observed here. Our data further implicate complex I as a focal point for regulation of mitochondrial quality and age‐related changes in mitochondrial function.

Increased reactive oxygen species and oxidative damage are frequently reported with age. We found that EDL displayed increased H_2_O_2_ production and a trend toward increased protein oxidative damage as measured by protein carbonylation. It is not likely a coincidence that respiratory complex I is a major source of reactive oxygen species, and this may be a direct result of the reduced respiratory efficiency in aged EDL. In contrast, SOL did not show increased H_2_O_2_ production or protein oxidative damage. However, the increased protein S‐glutathionylation does suggest a more oxidized redox environment. Thiol glutathionylation is now recognized as an important mechanism that not only helps prevent disruption of protein function by oxidative stress, but also can itself regulate protein function (Allen & Mieyal, 2012). In the aged SOL, increased protein S‐glutathionylation in the absence of oxidative protein damage, altered protein homeostasis, or a decline in respiratory efficiency is consistent with the hypothesis that protein S‐glutathionylation may be an early and protective response to redox stress.

Mice expressing mitochondrial‐targeted catalase reduce mitochondrial oxidative stress, delay age‐related pathology, and extend lifespan (Schriner *et al*., 2005; Treuting *et al*., 2008). Therefore, we used this model as a direct test of the role of mitochondrial oxidative stress in the loss of mitochondrial quality in aged skeletal muscle. Improved complex I respiratory capacity and efficiency in EDL of aged mCAT mice further support the hypothesis that oxidative stress plays a critical role in mitochondrial dysfunction with age.

**Figure 6:**
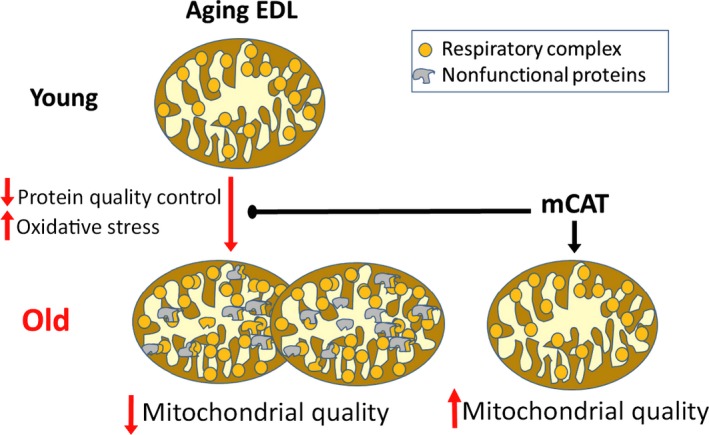
Schematic Model of Mitochondrial Dysfunction with Age. Mitochondrial protein quality control declines in susceptible tissue while oxidative stress increases with age. This leads to mitochondrial dysfunction presented by accumulation of mitochondrial respiratory proteins to compensate for decreased mitochondrial quality. Mitochondrial‐targeted antioxidant (mCAT) improves protein quality with age resulting in improved mitochondrial function.

Our data support an important role for the loss of mitochondrial proteostasis in reduced mitochondrial quality with age in the EDL. The loss of stoichiometry and lower mitochondrial efficiency due to the elevation of mitochondrial protein expression, particularly complex I, is indicative of lack of a coordinated response of the mitochondrial proteome to age‐induced stress in susceptible tissue. An important feature of this model is the longer half‐lives of mitochondrial proteins that results in accumulation of poorly functioning mitochondrial proteins that is prevented by improved mitochondrial antioxidant defense (Fig. 6). Thus, the decline in mitochondrial quality despite the conservation of respiratory capacity in permeabilized aged EDL highlights the importance of considering both quality (e.g., respiratory efficiency and H_2_O_2_ production) and capacity when examining age‐related mitochondrial dysfunction. The changes in protein homeostasis in the aged mitochondria, particularly in the EDL, suggest a breakdown of coordination of biosynthesis and protein recycling.

Ultimately, these data show tissue‐specific effects of age on mitochondrial protein homeostasis and function and implicate mitochondrial complex I as an especially important component. In summary, loss of protein homeostasis occurs with age, is convergent on mitochondria, specifically complex I, and is muscle type specific. These different effects of age warrant further study in how similar tissues with dissimilar mitochondrial content adapt to age and mitochondrial dysfunction. The tissue‐type specificity of age‐dependent modification of protein homeostasis and respiratory efficiency also suggest that therapeutic approaches may also be tissue type specific and targeted to optimizing mitochondrial electron transport system function.

## Experimental procedures

### Animal experiments

Animal experiments were performed with the approval of the Institutional Animal Care and Use Committee of the University of Washington. With the exception of mice used for isotope labeling for proteomics (described below), C57Bl/6J female mice (NIA) were maintained on standard rodent diet (5053, PicoLab). Food and water were available ad libitum in a vivarium with a 12‐h light/dark cycle at 22 °C.

### Muscle permeabilization

Mice were anaesthetized with 2,2,2‐tribromoethyl alcohol dissolved in tert‐amyl alcohol and skeletal muscle was carefully dissected, weighed, and placed into ice‐cold isolation buffer (100 mm Ca/EGTA, 5.8 mm NaATP, 6 mm MgCl, 20 mm Taurine, 15 mm phosphocreatine, 20 mm imidazole, 0.5 mm DTT, and 50 mm K‐MES, pH 7.1) as previously described (Siegel *et al*., 2012). Fascia, fat, and connective tissue were removed from muscle fiber bundles under a dissecting microscope, and muscle was carefully separated from surrounding fibers with forceps. Separated fiber bundles held together at either end by the tendons were then placed into permeabilization buffer (isolation buffer with 50 μg mL^−1^ saponin) and incubated on ice while gently shaking for 30–40 min. Muscle was then rinsed free of saponins in isolation buffer followed by incubation in respiration buffer on ice while shaking for at least 10 min (0.5 mm EGTA, 3 mm MgCl_2,_ 10 mm KH_2_PO_4_, 20 mm Hepes, 110 mm Sucrose, 1 g L^−1^ BSA, 20 mm Taurine, 20 mm K‐MES, pH ~7.1 with 5N KOH). Oxygen concentration was maintained > 200 μm to avoid oxygen diffusion limitations.

### Respirometry

Activities of respiratory complexes I, II, and IV were measured by monitoring the rate of oxygen consumption with an oxygen electrode (Oroboros Instruments, Innsbruck, Austria) in the presence of complex‐specific substrates. Rates were reported after substracting non‐mitochondrial oxygen consumption as previously described (Kruse *et al*., 2008). Briefly, leak respiration was determined in the presence of 10 mm glutamate, 5 mm pyruvate, and 2 mm malate in the absence of adenylates. To measure flux through complex I 2.5 mm ADP was added. To measure the quality of the mitochondria, the integrity of the outer membrane of mitochondria was tested by the addition of 10 μm cytochrome C. Any increase in flux would suggest that mitochondria were damaged and lost cytochrome C content during preparation, and that flux was then restored with cytochrome C addition. However, every muscle preparation passed this test as no increase in flux was observed. This was followed by the addition of 10 mm succinate to measure maximal flux (state 3) with the contribution of both complexes I and II. To measure maximal electron transport system (ETS) activity, we uncoupled oxygen flux from ATP synthase by titrating 0.5–3.5 μm CCCP to maximal flux. Rotenone (0.5 μm) was added to observe the contribution of complex II followed by 2.5 μm antimycin A to measure nonmitochondrial oxygen consumption. Complex IV was measured by the addition of 500 μm TMPD and 2 mm ascorbate followed by 1 mm KCN to subtract background oxygen consumption to account for TMPD auto‐oxidation. CIV activity was typically less than maximal when measured at this point presumably due to some degradation of activity over the course of experiment.

### H_2_O_2_ measurement

Amplex Red reagent was used to react with H_2_O_2_ producing the fluorescent resorufin. Muscle fibers were carefully dissected, separated, and permeabilized as described above for the respiration experiments. Reaction was initiated by the addition of succinate, and fluorescence (at Ex = 530 nm, Em = 590 nm) was recorded every 5 min for 30 min as previously described (Siegel *et al*., 2012).

### Tissue preparation and mitochondrial extraction

EDL and SOL muscles were dissected, and either flash‐frozen in liquid nitrogen or a mitochondrial extract derived from equivalent muscle weight/buffer volume was prepared as previously described (Siegel *et al*., 2011). Briefly, muscle tissue was mechanically disrupted in phosphate buffer, and mitochondria were enriched by dual‐step differential centrifugation and resuspended in appropriate buffer. Muscle samples for Western analyses were stored at −80°C until thawed into extraction buffer.

### Citrate synthase

Citrate synthase activity was measured by following reaction of 5′, 5′‐dithiobis 2‐nitrobenzoic acid with CoA thiol forming the yellow 2‐nitro‐5‐thiobenzoic acid (Sigma, St. Louis, MO, USA). The same mitochondrial homogenates used for mass spectroscopy were used for this assay.

### Protein carbonyl detection

Protein Oxidation Detection kit was used as recommended by the manufacturer (Millipore, Billerica, MA, USA). Briefly, muscle extracts were derivitized with 2,4‐dinitrophenylhydrazine and probed for protein carbonyls using an antibody for DNP.

### Western analyses

Voltage‐dependent anion channel 1 protein (VDAC1, Santa Cruz 8828), a major outer mitochondrial membrane component and surrogate marker for mitochondrial content, as well as various subunits of respiratory complexes (complex I (NdufB8), complex II (30 kDa SDHB), complex III (Core protein 2 UQRC2), and complex IV (alpha /MTCO1); Abcam 110413), were used to measure mitochondrial content. Samples were not heated to avoid degradation of ETS subunits. Protein S‐glutathionylation was detected by blocking free sulfhydryl groups using N‐ethylmaleimide and detection with an antibody (Abcam 19534 and Virogen 101‐A‐100). Actin content does not significantly change with age (Thompson *et al*., 2006), so either actin or total protein content was used to normalize protein load. Protein content was determined by densitometry using Bio‐Rad imaging hardware and Quantity One software (Hercules, CA, USA).

### Stable isotope labeling

After 13 weeks of complete diet on synthetic chow, mice were started on a leucine‐deficient synthetic diet (TD.09846, Harlan Teklad, Madison, WI) with the light leucine fully replaced by 11 g kg^−1^ of deuterated [5,5,5 – 2H^3^] – L – leucine (Cambridge Isotope Laboratory, Tewksbury, MA, USA). Four young (5 months old) and four old (27 months old) mice were euthanized for tissue collections and proteomics analysis at 3, 7, 12, and 17 days after switching to 2H^3^ – leucine diet as previously described (Dai *et al*., 2014).

### Mass spectroscopy and analysis

Mitochondria‐enriched fractions of muscle tissue were processed and trypsin digested, and LC‐MS/MS analysis performed with a Waters nanoAcquity UPLC and a Thermo Scientific LTQ‐FT. Consistent efficiency of mitochondrial enrichment in each sample group was confirmed by comparing citrate synthase activity between whole tissue homogenates and enriched samples as described above. Samples were loaded onto a 2 cm × 100 micrometers Kasil‐fitted trap column at a rate of 2 μL min^−1^ prior to peptide separations on a 35 cm × 75 micrometers Integra‐Frit (New Objective, Woburn, MA, USA), both of which were packed with 4 micrometers Jupiter C12 beads (Phenomenex, Torrance, CA, USA). Peptides were eluted using a twohour gradient to 35% acetonitrile in 0.1% formic acid at a flow rate of 0.25 μL min^−1^. The HPLC column was connected via a zero dead‐volume union to a 25‐micrometer ID electrospray emitter where the tip had been pulled to 10 micrometers (New Objective). The raw data from MS/MS are available at https://chorusproject.org/anonymous/download/experiment/-459620351802124584.

The Topograph software program was developed for the deconvolution and measurement of peptide isotopologue abundances from LC‐MS chromatograms using MS1 peak areas, and the calculation of peptide turnover rates, as previously described (Hsieh *et al*., 2012) ( http://proteome.gs.washington.edu/software/topograph/). After the percent of newly synthesized peptide was calculated for each of the multiple peptides that uniquely mapped to one protein, these values were plotted for each sample at each time point to generate an exponential curve following first‐order kinetics. Using a logarithmic transformation, the first‐order protein turnover rate (slope) was determined by linear regression (Hsieh *et al*., 2012).

Topograph, similar to the widely used Skyline platform (MacLean *et al*., 2010), uses a proven algorithm based on CRAWDAD ( http://pubs.acs.org/doi/abs/10.1021/ac701649e) to improve spectral alignment and peak integration (Hsieh *et al*., 2012; Schilling *et al*., 2012; Dai *et al*., 2014). Thus, for comparison of relative abundance between two experimental groups, we applied Topograph chromatogram alignment to correct chromatographic drift that may occur during the LC‐MS/MS and allowing comparisons of low abundance analytes that may be detected in only one but not the other samples. For peptides that were identified in one sample, the regression of the identified peptide's MS/MS scan number is used to estimate a window for the same peptide in the other samples and a matching chromatographic peak was identified within that time range.

Statistical analyses were performed using either Stata IC10, R or Bioconductor. Analyses used only peptides that mapped to a single protein. For the cases where a protein consisted of more than one peptide, statistical models were modified to appropriately account for the multiple peptides using a blocking factor. For each protein, we applied a nonlinear regression fit of first‐order exponential curves to the % newly synthesized protein using: y = 100 +  β1eαt. To determine whether the slopes α were statistically significantly different between experimental group, we used ANCOVA. Half‐lives were calculated according to first‐order kinetics: t_1/2_ = ln(2)/ slope.

For proteomics relative abundance data, statistically significant changes of proteins between experimental groups were determined using a linear model of peptide abundance to calculate fold changes of proteins between experimental groups in the same manner as a two‐sample t‐test using the R/Bioconductor software. The linear model gave *P*‐values that were adjusted for multiplicity with the Bioconductor package q‐value, which allows for selecting statistically significant genes while controlling the estimated false‐discovery rate.

The networks and canonical pathways were generated through the use of Ingenuity Pathway Analysis (IPA, Ingenuity Systems, www.ingenuity.com). A q < 0.05 was set to identify molecules whose expression was significantly differentially regulated. Canonical pathway analysis identified the pathways from the IPA library of canonical pathways that were significant to the dataset. The significance of the association between the data set and the canonical pathway was derived by taking a ratio of the number of molecules from the data set that map to the pathway divided by the total number of molecules present in the canonical pathway. The Fisher's exact test was used to calculate a *P*‐value reflecting the probability that the association between the mapped proteins in the dataset and the canonical pathway is explained by chance alone.

To compare mitochondrial to total protein abundance, we used an integrated database on mitochondrial proteins in yeast, man, mouse, and Neurospora (mitoP2, http://www.hsls.pitt.edu/obrc/index.php?page=URL1097158105) as a reference set of mitochondrial proteins.

Statistical analyses for nonproteomic data were performed using GraphPad Prism 6.0 (La Jolla, CA, USA) software. To determine differences between groups, two‐tailed student's t‐test were performed with significance defined as *P* < 0.05. Data are presented and means and standard error of the mean throughout.

## Conflict of interest

The authors declare that there are no competing interests.

## Author contributions

Experiments were designed by SEK and DJM. Proteomics approach was designed by PPK, MJM, and PSR. Experiments were carried out by SEK with the exceptions of stable isotope labeling by PPK, mass spectrometry by RJ, and assistance with data generation and heatmap production by NB. Statistical consultation and proteomics data generation provided by RPB. Data interpretation was carried out by SEK, DJM, and PSR. SEK and DJM wrote the manuscript.

## Supporting information


**Fig. S1** Protein half‐life is modified with age and is dependent on muscle type (B).
**Fig. S2** EDL and SOL Tissue Weights.
**Fig. S3** Minimal Inverse Correlation between Protein Abundance and Half‐life.
**Fig. S4** Oxidative State of Skeletal Muscle: Muscle Fiber H_2_O_2_ Production, Protein Carbonyl content and GSH‐modified Proteins.
**Table S1** Ingenuity Pathway Analysis of Proteins that Change Half‐Life with Age, q < 0.05, excluding pathways with <4 gene products that changed with age.
**Table S2** Abundance and Turnover of All Mitochondrial Proteins Detected in EDL and SOL.
**Table S3** Respiratory complex I changes of protein half‐lives with age, q < 0.05.
