# Supplementary material for: Age modifies respiratory complex I and protein homeostasis in a muscle type‐specific manner
Source: Aging Cell. 2015 Oct 25;15(1):89–99. doi: 10.1111/acel.12412 (PMC4717270; doi:10.1111/acel.12412)
Supplement: Supplementary file 1 — Fig. S1 Protein half‐life is modified with age and is dependent on muscle type (B). Fig. S2 EDL and SOL Tissue Weights. Fig. S3 Minimal Inverse Correlation between Protein Abundance and Half‐life. Fig. S4 Oxidative State of Skeletal Muscle: Muscle Fiber H2O2 Production, Protein Carbonyl content and GSH‐modified Proteins. Table S1 Ingenuity Pathway Analysis of Proteins that Change Half‐Life with Age, q < 0.05, excluding pathways with <4 gene products that changed with age. Table S2 Abundance and Turnover of All Mitochondrial Proteins Detected in EDL and SOL. Table S3 Respiratory complex I changes of protein half‐lives with age, q < 0.05. [file ACEL-15-089-s001.pdf]

Supplementary Figure 1

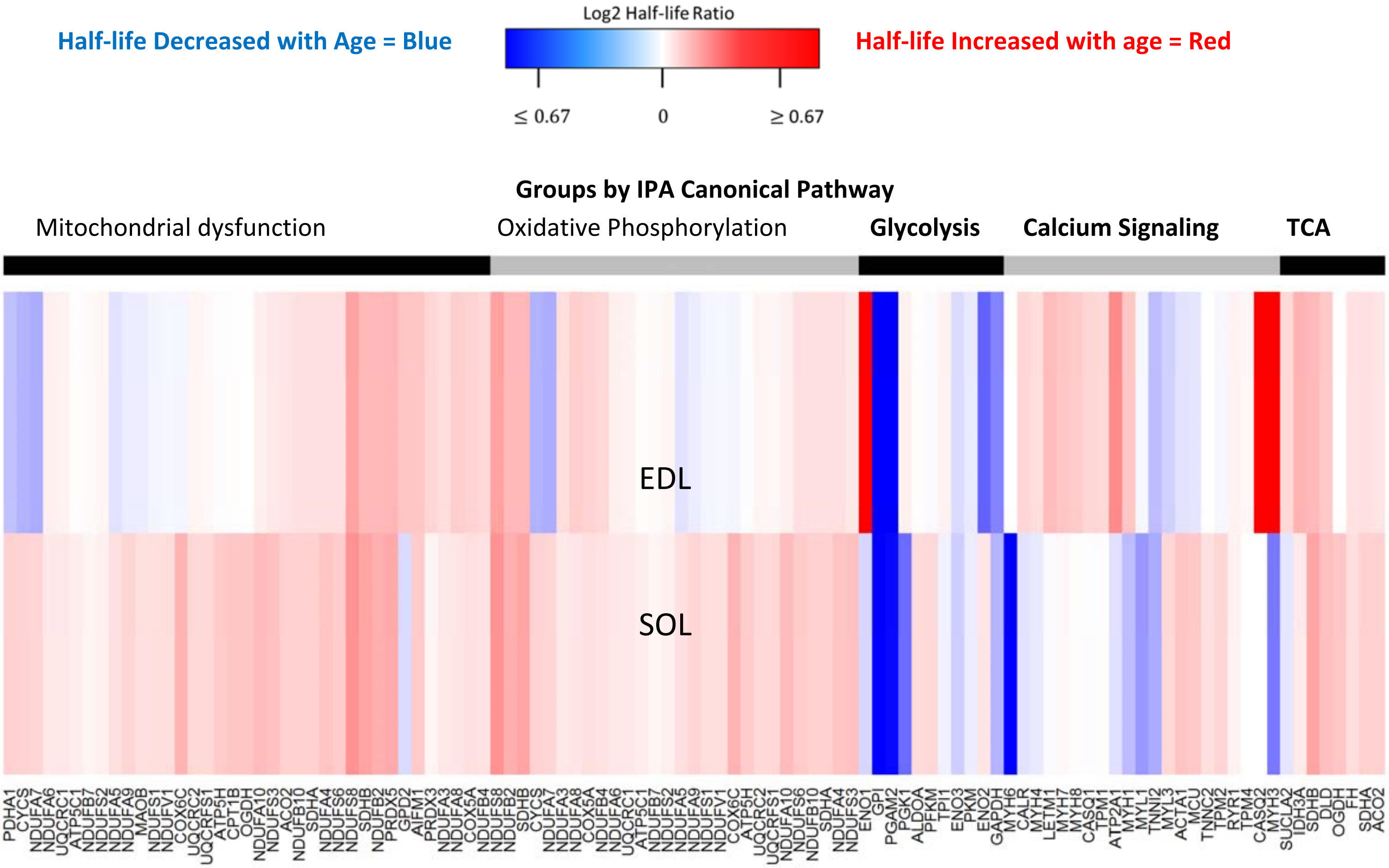

Supplementary Figure 2

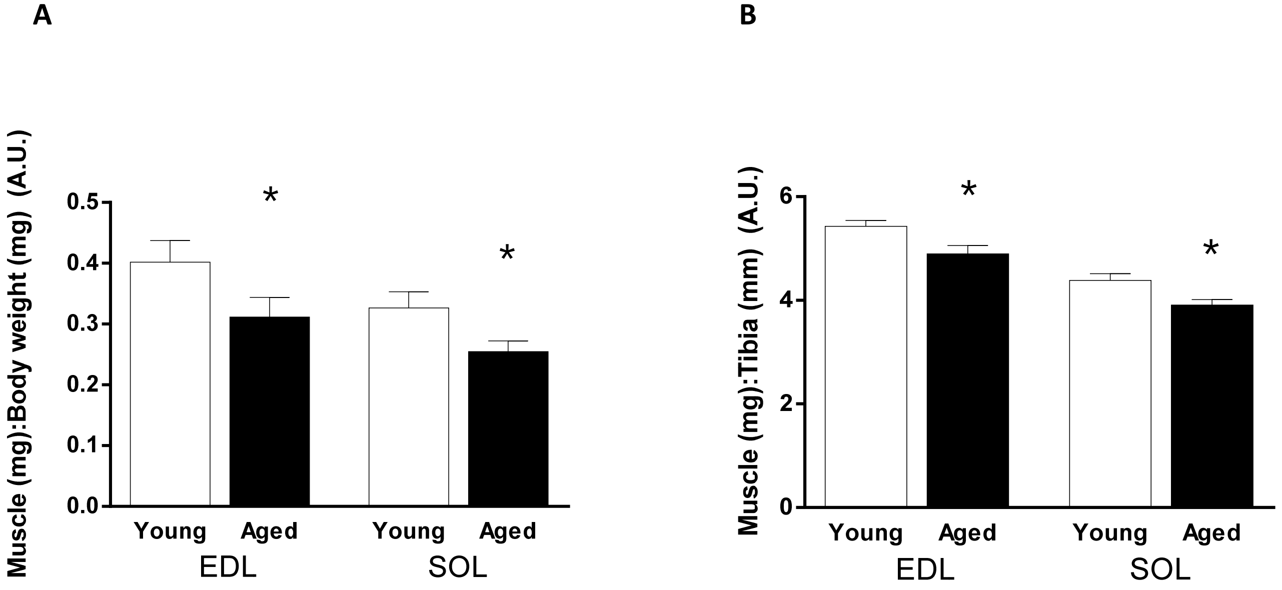

Supplementary Figure 3

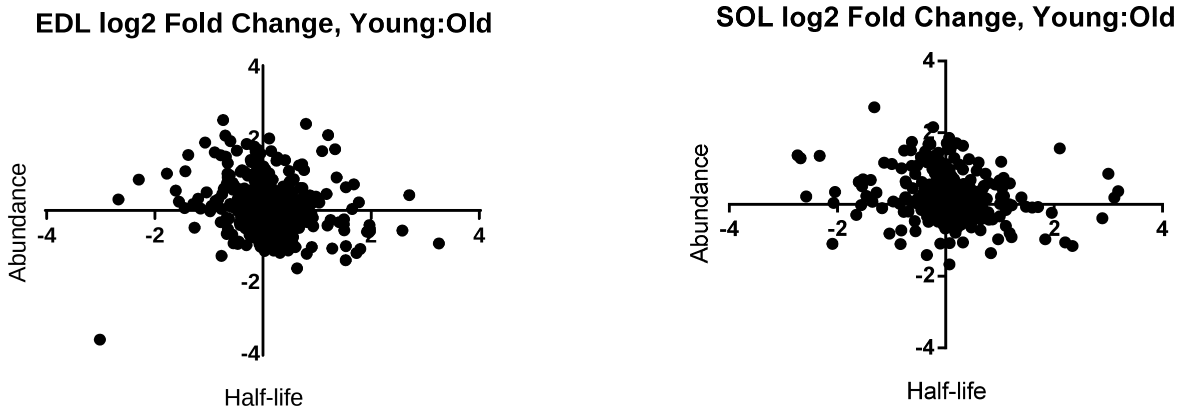

Supplementary Figure 4

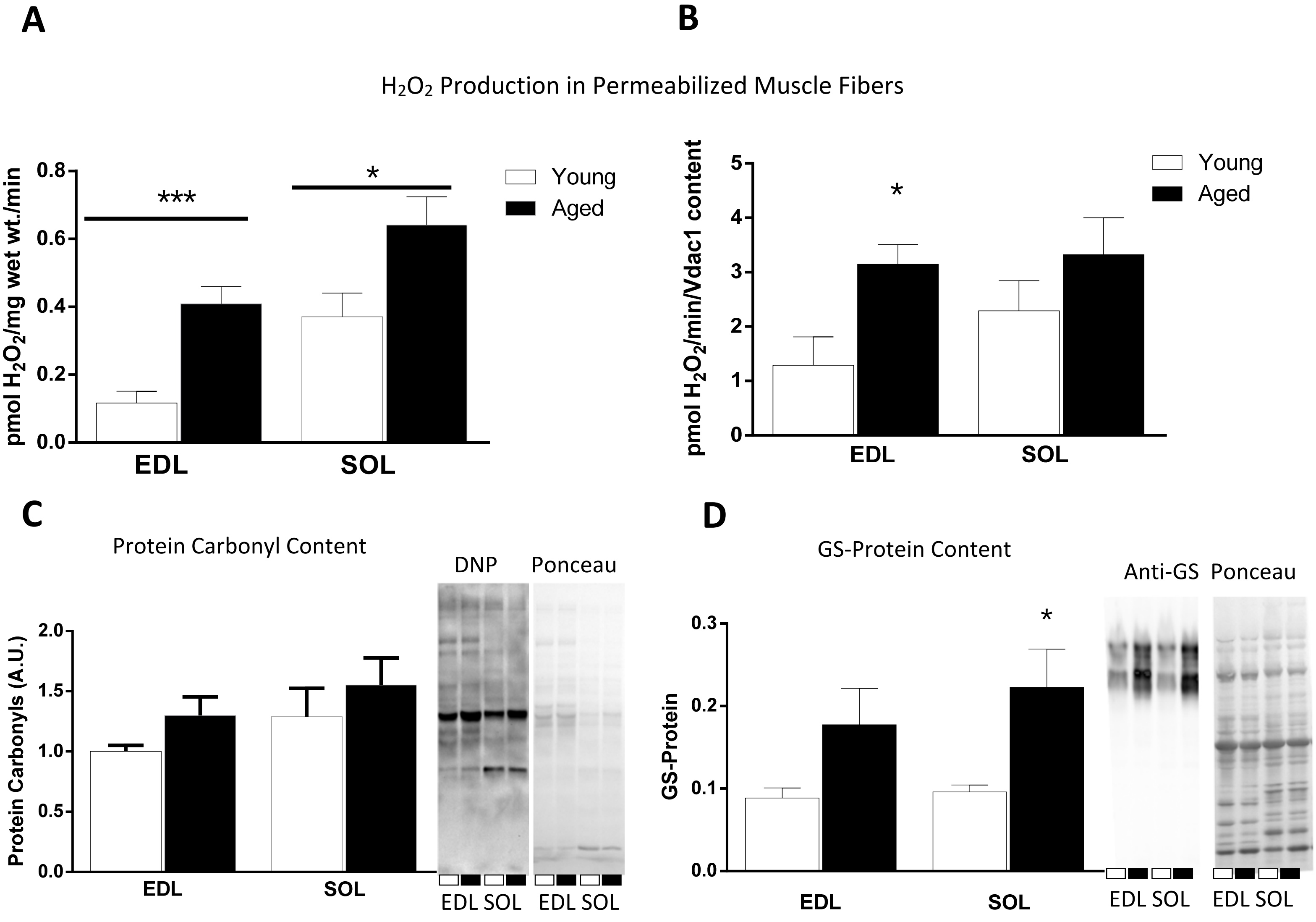

Supplemental Table 1. Ingenuity Pathway Analysis of Proteins that Change **Half-Life** with Age, $q < 0.05$, excluding pathways with less than 4 gene products that changed with age. Top five pathways not numbered, labelled in Supplemental Figure 1.

Mytochondrial Dysfunction
Oxidative Phosphorylation
Glycolysis
Calcium signaling
TCA

1. Fatty Acid β-oxidation I
2. Gluconeogenesis I
3. Isoleucine Degradation I
4. Acetyl-CoA Biosynthesis I (Pyruvate Dehydrogenase Complex)
5. Actin Cytoskeleton Signaling
6. Ketolysis
7. Tryptophan Degradation III (Eukaryotic)
8. Ketogenesis
9. Glutaryl-CoA Degradation
10. Valine Degradation I
11. Epithelial Adherens Junction Signaling
12. Cellular Effects of Sildenafil (Viagra)
13. L-cysteine Degradation III
14. Protein Kinase A Signaling
15. ILK Signaling
16. Mevalonate Pathway I
17. Regulation of Actin-based Motility by Rho
18. Creatine-phosphate Biosynthesis
19. 2-ketoglutarate Dehydrogenase Complex
20. Mitochondrial L-carnitine Shuttle Pathway
21. Amyotrophic Lateral Sclerosis Signaling
22. Superpathway of Geranylgeranyldiphosphate Biosynthesis I (via Mevalonate)
23. LXR/RXR Activation
24. RhoA Signaling
25. Tight Junction Signaling
26. PAK Signaling
27. Superpathway of Cholesterol Biosynthesis
28. Induction of Apoptosis by HIV1
29. Glycogen Degradation II
30. Agranulocyte Adhesion and Diapedesis
31. Paxillin Signaling
32. Glycogen Degradation III
33. Integrin Signaling
34. Caveolar-mediated Endocytosis Signaling
35. Phenylalanine Degradation IV (Mammalian, via Side Chain)

36. RhoGDI Signaling
37. TR/RXR Activation
38. Huntington's Disease Signaling
39. Virus Entry via Endocytic Pathways
40. AMPK Signaling
41. Clathrin-mediated Endocytosis Signaling
42. VEGF Signaling
43. Regulation of Cellular Mechanics by Calpain Protease
44. Myc Mediated Apoptosis Signaling
45. Tumoricidal Function of Hepatic Natural Killer Cells
46. Aldosterone Signaling in Epithelial Cells
47. Diphthamide Biosynthesis
48. Inosine-5'-phosphate Biosynthesis II
49. Glutamate Degradation II
50. Fatty Acid β-oxidation III (Unsaturated, Odd Number)
51. Aspartate Biosynthesis
52. Lipid Antigen Presentation by CD1
53. Remodeling of Epithelial Adherens Junctions

Supplementary Table 2. Abundance and Turnover of All Mitochondrial Proteins Detected in EDL and SOL. Raw data is available at https://chorusproject.org/anonymous/download/experiment/-459620351802124584.

EDL

		Abundance, OCL/YCL		Turnover, OCL	Turnover, YCL	
**UniProtKB**	**Gene ID**	**log2 fold change**	**q value**	**t/2 (d)**	**t/2 (d)**	**q value**
**Q9D7D1**	**2310014L17Rik**	**3.848151615**	**0.001755**			
**Q78IR3**	**4632415L05Rik**	**0.916776988**	**0.125963**	**17.51869**	**1.83239**	**0.117066**
**E9Q1N0**	**4932431P20Rik**	**-0.04631484**	**0.449507**	**10.80931**	**3.44114**	**0.142262**
**Q9JI39**	**Abcb10**	**1.157868658**	**0.105269**			
**Q9DBL9**	**Abhd5**	**0.17656744**	**0.432634**	**28.37282**	**29.66354**	**0.174989**
**Q8BWT1**	**Acaa2**	**0.822816746**	**1.55E-12**	**28.6908**	**27.33177**	**8.64E-05**
**Q8JZN5**	**Acad9**	**0.892299862**	**0.033224**	**15.14911**	**11.42597**	**0.224552**
**P51174**	**Acadl**	**0.944896825**	**7.53E-18**	**26.82589**	**24.04754**	**0.000403**
**P45952**	**Acadm**	**0.689557488**	**6.28E-05**	**14.96563**	**15.89809**	**0.016579**
**Q07417**	**Acads**	**0.442136503**	**0.106729**	**30.91578**	**37.5592**	**0.002889**

P50544	Acadvl	0.520943168	5.27E-08	20.13079	19.18991	4.80E-05
Q8QZT1	Acat1	0.774097321	1.57E-09	24.1048	24.45496	0.014493
Q99KI0	Aco2	0.661383143	5.54E-23	28.67233	27.33857	0.037194
Q9CQR4	Acot13	0.449031846	0.06056	31.0054	31.134	0.205217
Q9QYR9	Acot2	-1.02917388	0.014231	32.93888	51.82663	0.018071
Q9QXD1	Acox2	0.285584913	0.396066	16.85415	10.49653	0.17261
Q3UZN1	Acp2	-0.13294216	0.397862	6.65562	11.8982	0.153594
P41216	Acsl1	0.381221736	1.84E-10	26.52958	26.50976	0.006353
P68134	Acta1	-0.04824303	0.423061	667.50402	704.22962	0.04751
Q9JI91	Actn2	0.064158608	0.368006	33.56315	34.78294	3.91E-08
O88990	Actn3	-0.3870418	0.000341	35.69876	35.07336	2.37E-07
Q60936	Adck3	0.81087445	3.11E-07	4.53161	3.97263	0.024764
P54822	Adsl	-1.62048165	0.001067	30.57848	56.67255	0.045373
P28650	Adssl1	-0.87936609	0.004987	14.72618	9.91822	0.115739
Q8N9S3	Ahsa2	-0.94059026	0.13592	44.5312	41.3299	0.220595
P29699	Ahsg	0.285921606	0.397862	3.54905	2.10086	0.136709
Q9Z0X1	Aifm1	0.292432367	0.002118	35.52696	31.97343	3.38E-06
Q9R0Y5	Ak1	-1.3244723	8.06E-07	8.36674	13.12986	0.016923
P45376	Akr1b1	-1.27267937	2.74E-06	4.16152	4.30493	0.232684
P45377	Akr1b8	0.439829355	0.04204	69.17203	36.23405	0.128579
Q9D1F4	Akt1s1	-1.6535136	0.010797	1.69443	1.52331	0.003364
P07724	Alb	-0.8639387	3.61E-18	1.75132	1.54727	1.43E-10
P47738	Aldh2	1.124285866	2.62E-05	7.09831	6.93117	0.102775
Q8CHT0	Aldh4a1	0.492453738	0.002815	27.84027	34.92306	0.219322
Q9EQ20	Aldh6a1	0.875700756	3.68E-06	21.80229	22.6055	0.078292
P05064	Aldoa	-1.22988763	2.73E-35	18.83458	18.69682	0.117066
Q9D8C3	Alg13	0.665964457	0.369498			

**Q3V1D3**	**Ampd1**	**0.109785596**	**0.413057**	**17.93476**	**14.31743**	**0.131738**
**Q02357**	**Ank1**	**-0.7136407**	**0.016448**	**26.91753**	**19.59808**	**0.149524**
**G5E8K2**	**Ank3**	**0.026596111**	**0.450061**	**6.38655**	**3.61108**	**0.108984**
**Q505D1**	**Ankrd28**	**2.286440549**	**0.120575**			
**Q8K298**	**Anln**	**-1.68752918**	**0.396055**			
**P07356**	**Anxa2**	**0.448856257**	**0.021495**	**22.93265**	**19.51307**	**0.015724**
**P48036**	**Anxa5**	**-3.32637421**	**0.082798**			
**P14824**	**Anxa6**	**-0.47610242**	**0.03115**	**22.84334**	**23.5694**	**0.03328**
**Q9D7N9**	**Apmap**	**0.27575323**	**0.318517**	**17.46172**	**12.32794**	**0.138439**
**Q9WV35**	**Apobec2**	**-0.30275559**	**0.092532**	**22.36003**	**22.33878**	**0.020191**
**Q9DCZ4**	**Apoo**	**0.450788512**	**0.003637**	**24.96873**	**25.16683**	**0.054871**
**Q78IK4**	**Apool**	**0.517070004**	**0.009271**	**56.19034**	**87.95737**	**0.020191**
**Q61210**	**Arhgef1**	**-0.22207965**	**0.392144**	**2.71531**	**1.42947**	**0.136648**
**A1IGU4**	**Arhgef37**	**-1.54307289**	**9.09E-05**	**16.62442**	**28.53795**	**0.008139**
**Q8R5J9**	**Arl6ip5**	**0.783150862**	**0.037968**	**12.69907**	**10.89418**	**0.103123**
**Q8R2G4**	**Art3**	**1.114714981**	**0.192676**	**20.92893**	**18.17269**	**0.005951**
**Q8BSY0**	**Asph**	**-0.1417596**	**0.313165**	**150.01264**	**164.02308**	**0.256895**
**Q99MQ4**	**Aspn**	**0.320961212**	**0.327272**	**1116.40521**	**420.18103**	**0.18056**
**Q6PA06**	**Atl2**	**-0.28282576**	**0.16065**	**10.20724**	**10.08149**	**0.232525**
**Q8VDN2**	**Atp1a1**	**0.581497768**	**0.206947**			
**Q6PIE5**	**Atp1a2**	**-0.13011394**	**0.095937**	**8.79627**	**8.83533**	**1.33E-06**
**P14094**	**Atp1b1**	**-0.24994336**	**0.318659**	**13.80532**	**40.49864**	**0.032152**
**P14231**	**Atp1b2**	**-0.09820269**	**0.422175**	**9.48912**	**5.98272**	**0.072606**
**Q8R429**	**Atp2a1**	**0.359348204**	**0.025027**	**25.46336**	**25.46351**	**0.000773**
**F6RQN3**	**Atp2a1**	**-0.16328518**	**0.260012**	**8.73367**	**5.70912**	**0.158408**
**Q64518**	**Atp2a3**	**-0.16587871**	**0.268993**	**32462.67981**	**29738.33669**	**0.082057**
**Q03265**	**Atp5a1**	**0.575240776**	**1.63E-21**	**23.67048**	**22.36251**	**0.093851**

**P56480**	**Atp5b**	**0.821583714**	**2.82E-38**	**27.87435**	**25.85534**	**0.112848**
**Q91VR2**	**Atp5c1**	**0.197627522**	**0.112161**	**32.42169**	**32.25559**	**0.040739**
**Q9D3D9**	**Atp5d**	**0.69024186**	**2.23E-09**	**43.87984**	**40.94366**	**0.07839**
**P56382**	**Atp5e**	**0.733666876**	**1.82E-05**	**34.8515**	**32.27031**	**0.055444**
**Q9CQQ7**	**Atp5f1**	**0.063386402**	**0.40469**	**38.59314**	**36.70798**	**0.267487**
**Q9DCX2**	**Atp5h**	**0.38832725**	**7.58E-07**	**55.99089**	**56.39311**	**0.156069**
**Q06185**	**Atp5i**	**-0.26738244**	**0.27652**	**32.50667**	**28.90445**	**0.03778**
**P97450**	**Atp5j**	**0.752650722**	**0.019843**	**25.25942**	**24.97594**	**0.078237**
**Q9DB20**	**Atp5o**	**-0.02378535**	**0.435459**	**46.08229**	**46.58003**	**0.237063**
**A3FIN4**	**Atp8b5**	**0.686975963**	**0.026587**			
**Q8R087**	**B4galt7**	**0.492619934**	**0.377013**	**25.96543**	**2.31146**	**0.052609**
**Q8CGM1**	**Bai2**	**-0.57823704**	**0.302176**	**13.89871**	**16.03713**	**0.071808**
**Q8R1X0**	**BC022960**	**-0.10276278**	**0.441354**	**35.96512**	**53.56556**	**0.011837**
**Q61335**	**Bcap31**	**-1.0882857**	**0.052583**	**33.72224**	**42.92358**	**0.009383**
**P59017**	**Bcl2l13**	**0.336002298**	**0.044548**	**19.42889**	**12.98865**	**0.163276**
**Q80XN0**	**Bdh1**	**1.016501199**	**1.53E-14**	**26.57244**	**17.52813**	**0.033694**
**O08539**	**Bin1**	**0.231819284**	**0.040713**	**11.73169**	**11.88053**	**0.001319**
**O55003**	**Bnip3**	**-0.50987114**	**0.27652**	**6.24821**	**4.28508**	**0.120932**
**P18572**	**Bsg**	**-0.22295346**	**0.337735**	**9.89894**	**7.10389**	**0.128971**
**Q8CFE5**	**Btbd7**	**0.168581294**	**0.146939**			
**Q8R066**	**C1qtnf4**	**-0.8062065**	**0.089182**	**56.69229**	**40.21898**	**0.255102**
**Q8CG14**	**C1sa**	**0.858027435**	**0.001669**	**92.31808**	**87.49208**	**0.140008**
**P16015**	**Ca3**	**-0.13634573**	**0.202104**	**29.11773**	**31.72309**	**3.03E-05**
**Q02789**	**Cacna1s**	**-0.06140671**	**0.380286**	**42.33479**	**42.33369**	**0.001387**
**O08532**	**Cacna2d1**	**-0.27377322**	**0.008511**	**41.85696**	**35.73273**	**0.014493**
**Q8R3Z5**	**Cacnb1**	**-0.03697038**	**0.423553**	**25.13625**	**27.92952**	**0.045373**
**Q3UKW2**	**Calm1**	**0.878950348**	**0.056846**	**16.75557**	**11.47237**	**0.149524**

**P62204**	**Calm1**	**-0.28450621**	**0.081314**	**16.79868**	**15.80584**	**0.231509**
**P14211**	**Calr**	**-0.2795431**	**0.199197**	**14.04929**	**12.98644**	**0.024708**
**O35887**	**Calu**	**-0.57025985**	**0.125488**	**20.24093**	**18.99359**	**0.114045**
**P11798**	**Camk2a**	**-0.41076455**	**0.040713**	**23.4588**	**22.18677**	**0.089449**
**O09165**	**Casq1**	**-0.29440523**	**0.025076**	**146.72264**	**135.72205**	**7.71E-06**
**O09161**	**Casq2**	**-0.07527411**	**0.379416**	**25.91207**	**16.32853**	**0.207895**
**P49817**	**Cav1**	**-0.62816284**	**0.005086**	**19.37055**	**11.66465**	**0.220596**
**Q9DA08**	**Ccdc101**	**-0.05993103**	**0.419058**	**1.82494**	**4.06492**	**0.067144**
**Q3TMW1**	**Ccdc102a**	**-1.66966145**	**0.040713**	**21.23864**	**29.65716**	**0.011837**
**Q3UX62**	**Ccdc114**	**0.017755487**	**0.448741**			
**Q810T2**	**Ccnb3**	**0.24765358**	**0.374172**	**17.70248**	**18.28798**	**0.050339**
**Q08857**	**Cd36**	**0.637706388**	**1.12E-06**	**7.64877**	**7.60414**	**0.014466**
**Q7TT50**	**Cdc42bpb**	**-0.21235756**	**0.374172**	**6.81604**	**7.36674**	**0.146012**
**Q6A068**	**Cdc5l**	**1.040975826**	**0.103761**			
**Q9WTR5**	**Cdh13**	**0.358394955**	**0.294979**			
**Q9JKC6**	**Cend1**	**0.806647322**	**0.225097**			
**Q6A065**	**Cep170**	**0.989909256**	**0.294979**	**37.14395**	**12.79649**	**0.219322**
**Q8VCT4**	**Ces1d**	**6.283126071**	**0.035776**			
**P45591**	**Cfl2**	**-1.12622512**	**0.03115**	**31.04498**	**40.55908**	**0.242216**
**Q6AW69**	**Cgnl1**	**-2.10646395**	**0.008057**	**140.0518**	**60.67633**	**0.19833**
**Q9CRB9**	**Chchd3**	**0.016069058**	**0.432634**	**31.33671**	**30.2247**	**8.10E-05**
**P26339**	**Chga**	**0.428808582**	**0.261108**	**16.95202**	**12.43301**	**0.208378**
**Q8BIW9**	**Chtf18**	**-0.7232379**	**0.388937**			
**Q91WS0**	**Cisd1**	**0.170132368**	**0.301513**	**30.12945**	**30.27516**	**0.045373**
**B1AR13**	**Cisd3**	**0.354492989**	**0.109844**	**9.38011**	**10.5831**	**0.058229**
**P07310**	**Ckm**	**-1.70492273**	**1.77E-46**	**36.04167**	**38.67093**	**0.000318**
**Q6P8J7**	**Ckmt2**	**0.538502297**	**2.23E-09**	**32.13635**	**32.7595**	**0.000249**

Q68FD5	Cltc	-2.10968436	0.27652			
Q8R4N0	Clybl	0.494731185	0.021495	26.59288	24.66505	0.092644
P16330	Cnp	-0.49956393	0.327272	55.16488	43.17514	0.131738
P11087	Col1a1	-0.08087285	0.431639	177.45199	120.31821	0.091606
Q01149	Col1a2	-0.42684196	0.317798	68.03491	10.40052	0.182284
P28481	Col2a1	-0.31223426	0.392341	1.01989	6.50964	0.125688
Q8BMS4	Coq3	0.669517879	0.001447	30.94242	45.87535	0.073654
Q8K1Z0	Coq9	0.932164072	9.02E-13	17.8146	18.55854	0.032017
P19783	Cox4i1	0.545409151	0.000102	29.47529	30.25528	0.054871
P12787	Cox5a	0.30317966	0.004503	23.06849	21.29262	0.002979
P19536	Cox5b	0.217490859	0.10527	26.86081	25.39388	0.00075
P43024	Cox6a1	0.35974642	0.149833	16.4827	11.79198	0.13879
P43023	Cox6a2	0.320629326	0.121312	9.65687	8.8911	0.089449
P56391	Cox6b1	0.133146855	0.316188	33.46993	32.8591	0.182201
Q9CPQ1	Cox6c	0.426629484	0.115432	28.34736	28.8973	0.089449
P56392	Cox7a1	0.546835125	0.001361	19.56853	20.33306	0.166999
P48771	Cox7a2	0.499562744	6.44E-05	9.18097	8.64354	0.213292
Q0VE82	Cpne7	-0.49241579	NA			
Q924X2	Cpt1b	0.212822074	0.053719	16.8838	16.75665	0.008389
P52825	Cpt2	0.566929789	2.70E-05	19.5557	20.12033	0.001259
P47934	Crat	0.748583762	0.000281	27.53087	24.63699	0.020007
Q9D2A5	Creb3l4	-0.33016548	0.27652	69.88498	160.51133	0.162067
P23927	Cryab	-0.4251165	0.073305	7.61356	7.79589	0.010247
Q9CZU6	Cs	0.824916259	1.87E-13	29.70763	26.16544	0.237301
Q99388	Csprs	0.793101118	0.329547			
P18242	Ctsd	0.41734123	0.144012	7.35272	5.64726	0.13059
Q9CQX2	Cyb5b	0.129678117	0.402702			

**Q9DB73**	**Cyb5r1**	**-1.00306925**	**0.103761**	**19.57788**	**23.27454**	**0.163276**
**Q9D0M3**	**Cyc1**	**0.171855898**	**0.151151**	**42.42943**	**42.9751**	**0.054871**
**P62897**	**Cycs**	**0.256841312**	**0.026587**	**34.45221**	**39.76666**	**0.00057**
**Q9D172**	**D10Jhu81e**	**0.489904812**	**0.001347**	**27.02586**	**26.76835**	**0.24083**
**P31786**	**Dbi**	**-0.28438402**	**0.306542**	**33.08517**	**27.55123**	**0.0099**
**Q8BGW4**	**Dcaf12l2**	**0.743161275**	**0.003637**	**20.21575**	**17.30207**	**0.106426**
**Q9JLM8**	**Dclk1**	**-0.20077225**	**0.37585**	**23.1919**	**13.24478**	**0.065037**
**P28654**	**Dcn**	**0.306247426**	**0.086559**	**8.44055**	**6.52148**	**0.246928**
**Q9CQ62**	**Decr1**	**0.916125553**	**1.46E-11**	**28.08232**	**22.06892**	**0.000549**
**P31001**	**Des**	**-0.52012716**	**0.001892**	**9.53248**	**8.66219**	**0.119491**
**Q99LB2**	**Dhrs4**	**0.521936182**	**0.256352**	**49.71163**	**33.15298**	**0.244956**
**Q8CHS7**	**Dhrs7c**	**-0.46068927**	**9.09E-05**	**44.84687**	**43.55004**	**0.001277**
**Q8BMF4**	**Dlat**	**0.678154366**	**2.43E-20**	**52.37012**	**56.4308**	**0.041601**
**O08749**	**Dld**	**0.733714485**	**1.50E-16**	**39.58827**	**35.89905**	**0.002979**
**Q9D2G2**	**Dlst**	**0.622233114**	**2.25E-06**	**49.57198**	**50.65133**	**0.055953**
**Q8VHE6**	**Dnah5**	**0.756383574**	**0.155525**			
**Q9QYI6**	**Dnajb9**	**0.03644714**	**0.422175**	**10.00683**	**10.94093**	**0.003765**
**O08553**	**Dpysl2**	**-0.77092412**	**0.292117**	**3.16641**	**4.66555**	**0.043481**
**Q8BLI4**	**Dse**	**0.482999634**	**0.115044**	**3.91716**	**3.90596**	**0.050097**
**Q9D2N4**	**Dtna**	**-0.07438158**	**0.428553**	**46.70058**	**34.35313**	**0.11005**
**Q8BH95**	**Echs1**	**0.673803387**	**0.000451**	**35.9576**	**42.66115**	**0.045373**
**P42125**	**Eci1**	**0.193295744**	**0.288499**	**27.75892**	**27.06423**	**0.187922**
**Q9WUR2**	**Eci2**	**-0.30881662**	**0.291539**	**9.82185**	**9.27829**	**0.041194**
**P62631**	**Eef1a2**	**0.422921166**	**0.071043**	**18.64758**	**18.22**	**0.100242**
**Q9D8N0**	**Eef1g**	**1.543839059**	**0.329619**			
**P58252**	**Eef2**	**0.115105219**	**0.389381**	**9.24368**	**9.96715**	**0.010652**
**Q8BH64**	**Ehd2**	**1.055610117**	**0.143446**			

**Q99LC8**	**Eif2b1**	**-0.76102718**	**0.380286**			
**P17182**	**Eno1**	**-0.33044778**	**0.317798**	**47.4695**	**28.86611**	**0.031098**
**P17183**	**Eno2**	**0.334056022**	**0.316698**	**27.83512**	**36.92844**	**0.010231**
**P21550**	**Eno3**	**-1.11046533**	**2.63E-18**	**33.77856**	**36.11618**	**0.036517**
**O70318**	**Epb41l2**	**-0.73160716**	**0.195248**	**20.21421**	**6.29452**	**0.163376**
**Q99M71**	**Epdr1**	**-0.33155685**	**0.141722**	**6.568**	**9.0578**	**0.005265**
**Q8VEH5**	**Epm2aip1**	**-0.08777966**	**0.42479**			
**Q99K30**	**Eps8l2**	**-1.08535955**	**0.157147**	**8.80018**	**11.71597**	**0.01905**
**O08580**	**Esrra**	**0.127269421**	**NA**			
**Q99LC5**	**Etfa**	**0.39667027**	**0.000322**	**27.72398**	**28.71458**	**4.20E-05**
**Q9DCW4**	**Etfb**	**0.656190238**	**3.41E-10**	**25.19793**	**25.19052**	**0.015724**
**Q921G7**	**Etfdh**	**0.327589447**	**0.001813**	**30.35514**	**30.13508**	**0.024708**
**Q9DCM0**	**Ethe1**	**0.479950058**	**0.317798**	**20.31934**	**48.74205**	**0.001259**
**Q8BI71**	**Exoc3l1**	**-0.84014212**	**0.198407**	**67.99245**	**104.00921**	**0.098705**
**Q8R313**	**Exoc6**	**-0.09173561**	**0.42479**	**21.26444**	**22.91205**	**0.003957**
**Q3T9V5**	**F830112A20Rik**	**0.11097464**	**0.449308**			
**P55050**	**Fabp2**	**-0.10348224**	**0.422175**	**9.95312**	**12.18838**	**0.156783**
**P11404**	**Fabp3**	**-0.61347445**	**0.089182**	**32.48961**	**41.29411**	**0.004067**
**P04117**	**Fabp4**	**0.319150703**	**0.182522**	**13.9472**	**14.8414**	**0.138196**
**O08716**	**Fabp9**	**0.402737228**	**0.299987**	**3.26183**	**0.83168**	**0.208378**
**Q8R0F8**	**Fahd1**	**0.246764241**	**0.309859**	**28.57314**	**30.52417**	**0.055953**
**Q3U2I3**	**Fam160a2**	**-1.68400123**	**0.212035**			
**Q9D6U8**	**Fam162a**	**0.348613434**	**0.253747**	**15.21763**	**18.61288**	**0.071454**
**Q14CH0**	**Fam171b**	**0.335676151**	**0.418953**	**23.48679**	**31.65347**	**0.02829**
**Q61554**	**Fbn1**	**-0.15798393**	**0.422175**	**121.80179**	**66.78587**	**0.189727**
**Q78JE5**	**Fbxo22**	**0.429728486**	**0.159818**	**8.99556**	**9.35066**	**0.133926**
**Q8BI58**	**Fbxw26**	**-0.58151752**	**0.325158**			

**P70451**	**Fer**	**-0.57020043**	**0.322599**			
**P97807**	**Fh**	**0.711163496**	**1.55E-12**	**29.93473**	**28.37366**	**1.78E-07**
**P97447**	**Fhl1**	**-0.37294439**	**0.126642**	**31.03082**	**30.07294**	**0.189727**
**Q9CS72**	**Filip1**	**1.345762514**	**0.018876**			
**Q91V79**	**Fitm1**	**-0.45373426**	**0.027981**	**7.27211**	**7.84306**	**0.050339**
**Q8VHX6**	**Flnc**	**-0.13640428**	**0.379563**	**6.64824**	**7.15298**	**0.013831**
**Q8R2I0**	**Foxe1**	**-0.19342293**	**0.43295**	**15.79042**	**16.41716**	**0.151153**
**E9Q8I9**	**Fry**	**-0.58527749**	**0.328384**			
**Q32M02**	**Fscn2**	**-0.16002891**	**0.402702**	**18.68141**	**12.70371**	**0.157725**
**P16858**	**Gapdh**	**-1.05648691**	**3.81E-10**	**33.45915**	**41.79909**	**0.036517**
**Q01721**	**Gas1**	**0.482164358**	**0.18945**	**1.30774**	**0.89924**	**0.125204**
**P17679**	**Gata1**	**-0.42265028**	**0.292117**	**1.62743**	**0.89015**	**0.015186**
**O55126**	**Gbas**	**0.505867983**	**0.001483**	**27.62242**	**27.27658**	**0.003364**
**Q5SV77**	**Ggnbp2**	**-0.51199421**	**0.392144**			
**Q9CR36**	**Gkn1**	**1.57806247**	**0.126946**			
**Q80Y14**	**Glrx5**	**0.396755536**	**0.306542**	**8.11312**	**7.26176**	**0.091894**
**P26443**	**Glud1**	**0.594416913**	**4.78E-05**	**26.50149**	**21.99404**	**0.131738**
**Q3UN64**	**Gm10564**	**0.55677665**	**0.37773**	**10.21313**	**1.70551**	**0.131392**
**Q3TNV0**	**Gm10941**	**-1.80838559**	**0.268759**			
**D3Z1B2**	**Gm4953**	**0.080552335**	**0.379563**	**8.76812**	**4.71141**	**0.138439**
**Q3ULJ8**	**Gm4981**	**-1.2787151**	**0.047681**			
**D3Z1Y8**	**Gm6483**	**-0.18569374**	**0.38593**	**3.80882**	**5.80822**	**0.146012**
**F6QVC4**	**Gm9732**	**0.006837049**	**0.462271**	**64.68238**	**49.26446**	**0.203751**
**Q9DCZ1**	**Gmpr**	**-0.74283261**	**0.049875**	**10.88105**	**7.76551**	**0.252248**
**Q692V3**	**Gnn**	**0.286582798**	**0.380597**			
**P05201**	**Got1**	**-0.95573703**	**1.41E-11**	**19.73382**	**21.41121**	**0.004589**
**P05202**	**Got2**	**0.83954027**	**1.43E-20**	**35.25043**	**32.36124**	**0.114912**

**P13707**	**Gpd1**	**-0.84001079**	**0.050125**	**14.83781**	**16.55911**	**0.067251**
**Q64521**	**Gpd2**	**-0.1981752**	**0.012022**	**22.07906**	**19.91126**	**0.158669**
**P06745**	**Gpi**	**-0.7666232**	**0.000155**	**20.10227**	**31.8202**	**4.66E-06**
**Q8C010**	**Gpr61**	**0.1742562**	**0.413057**	**26.09302**	**27.96602**	**0.170478**
**Q03391**	**Grin2d**	**0.604529007**	**0.20234**	**8.33901**	**2.14106**	**0.222511**
**P10649**	**Gstm1**	**-0.66828249**	**0.245211**	**49.71659**	**38.27151**	**0.109494**
**P52785**	**Gucy2e**	**-1.70911357**	**0.021688**	**6.00167**	**2.38161**	**0.164832**
**Q9Z1E4**	**Gys1**	**-0.42619476**	**0.202925**	**10.27951**	**9.0383**	**0.103148**
**Q31168**	**H2-D1**	**0.482056042**	**0.266617**			
**Q61425**	**Hadh**	**0.701121465**	**9.04E-07**	**25.87742**	**23.5085**	**0.000112**
**Q8BMS1**	**Hadha**	**0.69266411**	**4.90E-25**	**29.10429**	**27.41581**	**9.17E-09**
**Q99JY0**	**Hadhb**	**0.540304918**	**2.84E-08**	**16.75737**	**16.87112**	**0.001386**
**P01942**	**Hba**	**-1.78424243**	**0.002468**	**30.93061**	**34.52654**	**0.11133**
**P02088**	**Hbb-b1**	**-0.15084259**	**0.402702**	**35.6782**	**37.08862**	**0.050097**
**Q7TQ32**	**Hfe2**	**-0.0710474**	**0.437693**	**26.9783**	**73.51811**	**0.000682**
**Q9D1G3**	**Hhatl**	**-0.00447306**	**0.45269**	**70.01695**	**66.48876**	**1.03E-05**
**Q14DK5**	**Hhipl1**	**0.20280211**	**0.294979**	**6.06097**	**4.1659**	**0.04809**
**Q99L13**	**Hibadh**	**1.067646201**	**0.013052**	**28.2916**	**8.1198**	**0.133161**
**P43274**	**Hist1h1e**	**0.361656831**	**0.327272**	**101.46369**	**100.98719**	**0.222868**
**P62806**	**Hist1h4a;**	**-0.45704645**	**0.080113**	**64.74078**	**75.37006**	**0.012796**
**Q6PG16**	**Hjurp**	**1.831256681**	**0.256352**			
**P63158**	**Hmgb1**	**0.136812452**	**0.405296**	**25.59071**	**16.55928**	**0.067251**
**Q01822**	**Hoxd1**	**0.004722946**	**0.462271**	**1.30384**	**2.53727**	**0.146012**
**Q6VYH9**	**Hsh2d**	**0.591806261**	**0.020888**	**24.13495**	**22.01225**	**0.097581**
**P20029**	**Hspa5**	**-0.207322**	**0.232485**	**19.30292**	**19.57937**	**0.217281**
**P63017**	**Hspa8**	**-0.19506523**	**0.392144**	**4.61136**	**6.26313**	**0.01539**
**P38647**	**Hspa9**	**0.441293303**	**0.02496**	**7.15463**	**7.56362**	**0.001821**

**P63038**	**Hspd1**	**0.578537286**	**4.18E-07**	**25.65017**	**22.87061**	**0.0099**
**Q64433**	**Hspe1**	**0.759344706**	**2.75E-07**	**21.52327**	**20.41769**	**0.249615**
**P54071**	**Idh2**	**0.917617717**	**1.18E-10**	**33.23868**	**26.28477**	**0.000901**
**Q9D6R2**	**Idh3a**	**0.456028703**	**0.070565**	**23.05983**	**20.0894**	**0.026176**
**Q91VA7**	**Idh3b**	**0.583740151**	**1.98E-07**	**18.06517**	**16.49508**	**0.129962**
**P70404**	**Idh3g**	**0.672484802**	**0.000142**	**22.42378**	**20.7328**	**0.129847**
**Q64282**	**Ifit1**	**0.406279021**	**0.279299**	**8.00675**	**10.70198**	**0.237341**
**Q8CAQ8**	**Immt**	**0.226554239**	**0.017166**	**29.46698**	**27.71899**	**0.000412**
**P85094**	**Isoc2a**	**0.269291038**	**0.118217**			
**Q00651**	**Itga4**	**0.232248324**	**0.409376**	**574.63114**	**402.37454**	**0.048706**
**Q9JHI5**	**Ivd**	**0.446941993**	**0.042344**	**20.96754**	**21.65958**	**0.239872**
**Q9ERI5**	**Jmjd6**	**1.190037965**	**0.377013**			
**Q9ET80**	**Jph1**	**0.032671079**	**0.422175**	**23.77217**	**21.596**	**0.202653**
**Q9ET78**	**Jph2**	**-0.64667786**	**0.022142**	**34.5443**	**37.53506**	**0.000691**
**Q3MI48**	**Jsrp1**	**0.018455625**	**0.447894**	**16.54781**	**19.41488**	**0.143294**
**Q02257**	**Jup**	**-0.02125565**	**0.454553**			
**Q9D783**	**Kbtbd5**	**0.953626509**	**0.168792**	**16.0799**	**10.99556**	**0.117498**
**P59111**	**Kcnh8**	**-0.44811147**	**NA**			
**Q61743**	**Kcnj11**	**0.235990371**	**0.392144**	**241.80916**	**279.87541**	**0.114045**
**Q9DCV6**	**Kiaa0141**	**1.118293285**	**0.253747**			
**Q8BGX7**	**Kiaa0146**	**0.191090217**	**0.390447**	**0.7548**	**0.63295**	**0.002313**
**Q7TQE7**	**Kiaa0895**	**0.607330989**	**0.328835**	**41.83874**	**37.46612**	**0.183589**
**Q8K2Q9**	**Kiaa1598**	**-0.1701929**	**0.36618**	**15.57584**	**14.00382**	**0.027433**
**E9Q4K7**	**Kif13b**	**0.936783179**	**0.317798**			
**Q7TNC6**	**Kif26b**	**0.022135281**	**0.458227**	**3.41392**	**3.65341**	**0.157725**
**Q922S8**	**Kif2c**	**-0.65138275**	**0.397801**	**439.69049**	**151.77535**	**0.027977**
**Q8BUL5**	**Klhl7**	**1.204510367**	**0.001128**	**40.9425**	**23.36342**	**0.034494**

**P02535**	**Krt10**	**0.644777644**	**0.027557**	**83.39354**	**85.90085**	**0.002329**
**Q61781**	**Krt14**	**1.383800567**	**0.199197**	**130.58721**	**45.14271**	**0.188578**
**Q61782**	**Krt14**	**0.866618719**	**0.27652**			
**Q9Z2K1**	**Krt16**	**0.486757891**	**0.369458**	**54.16129**	**40.35841**	**0.104494**
**Q9QWL7**	**Krt17**	**0.166285867**	**0.397801**	**36.43145**	**36.47176**	**0.02684**
**Q6IFX2**	**Krt42**	**0.961654625**	**0.079524**	**59.47857**	**73.36733**	**0.015005**
**Q922U2**	**Krt5**	**0.863632997**	**0.177954**	**80.85066**	**110.03193**	**0.05033**
**P50446**	**Krt6a**	**0.485108533**	**0.36053**	**199.80142**	**125.79809**	**0.145003**
**Q6IFZ6**	**Krt77**	**-1.85894042**	**0.313165**	**94.31024**	**116.19298**	**0.199292**
**Q8VED5**	**Krt79**	**0.483458478**	**0.317798**	**204.60001**	**241.9265**	**0.031098**
**Q9CXF0**	**Kynu**	**-0.55467652**	**0.325158**	**10.40738**	**31.81505**	**0.033392**
**P11438**	**Lamp1**	**0.156480364**	**0.379416**	**4.65637**	**3.24961**	**0.203542**
**Q9JKS4**	**Ldb3**	**-0.12778721**	**0.377013**	**30.72267**	**28.99333**	**0.153594**
**E9PYJ9**	**Ldb3**	**-0.82713097**	**0.071567**	**32.65087**	**29.38709**	**0.154304**
**P06151**	**Ldha**	**-1.35737848**	**3.72E-11**	**21.94889**	**25.46777**	**0.001572**
**P16125**	**Ldhb**	**-1.29312605**	**0.000203**	**46.44472**	**28.05306**	**0.119491**
**Q9Z2I0**	**Letm1**	**0.405634031**	**0.008346**	**16.15851**	**14.29688**	**0.040739**
**Q91VD1**	**Lgals12**	**0.552853765**	**0.051695**	**23.09738**	**8.14915**	**0.023909**
**Q8C129**	**Lnpep**	**-0.35429143**	**0.2103**	**1.55026**	**1.27653**	**0.117066**
**Q505F5**	**Lrrc47**	**-0.30997842**	**0.258651**	**47.10004**	**83.65965**	**0.014493**
**Q8K0B3**	**Lrrc66**	**1.097719881**	**NA**			
**Q8K2F8**	**Lsm14a**	**0.869591193**	**0.143791**	**5.48366**	**4.39145**	**0.123658**
**P97412**	**Lyst**	**0.801678895**	**0.316698**			
**Q9QXZ0**	**Macf1**	**-0.6291201**	**0.202104**	**20.45725**	**12.65594**	**0.11005**
**Q922B1**	**Macrod1**	**0.285535335**	**0.033191**	**23.45914**	**21.34698**	**0.021081**
**Q9WTX8**	**Mad1l1**	**3.593876595**	**0.186471**	**4.95182**	**40.10171**	**0.075721**
**Q8BW75**	**Maob**	**-1.58830789**	**0.020546**	**8.17872**	**8.45197**	**0.001902**

P27546	Map4	-0.03974965	0.437693	4.08085	2.73634	0.02829
P04247	Mb	-0.70986382	5.05E-05	30.56326	29.87096	0.202566
P04370	Mbp	0.30054081	0.11198	81.41925	66.45238	0.027143
Q3UMR5	Mcu	0.429873396	0.23317	23.52866	24.68317	0.00434
P14152	Mdh1	-0.99411564	6.44E-05	18.91975	21.18706	0.050288
P08249	Mdh2	0.854595495	1.13E-23	34.36091	32.13178	0.242073
Q8BMF3	Me3	0.329381059	0.27652	10.10356	7.75178	0.160266
Q9DCS3	Mecr	0.342712746	0.20234	41.78947	29.73554	0.216574
O88559	Men1	-0.59455016	0.226844	0.76694	1.26467	0.016985
Q811U4	Mfn1	-0.89340018	0.181723	18.21799	19.9615	0.000495
A2AWL7	Mga	-0.40827657	0.226466	7.46315	8.99463	0.247983
Q8BI84	Mia3	0.570234057	0.313165			
Q8BMD7	Morc4	-0.13842206	NA			
Q8VEL0	Mospd1	-0.12033956	0.428949	26.06345	50.52016	0.053216
P56379	Mp68	0.226284747	0.397801	23.14988	35.77272	0.158463
P63030	Mpc1	0.211806229	0.327272	9.97864	9.50607	0.189727
Q99J99	Mpst	0.509378119	0.007874	18.52343	16.33227	0.03328
P27573	Mpz	-0.13560805	0.392144	77.35721	59.57552	0.072606
Q3UR85	MRF	-0.11779318	0.422175	111.08427	137.5077	0.190375
Q9CY16	Mrps28	-0.63109598	0.231182			
Q61733	Mrps31	-0.04876256	0.402702	4.24611	7.23843	0.110475
Q9CQX8	Mrps36	0.456344223	0.010662	25.63603	25.58749	0.065037
P03930	Mtatp8	0.496795376	0.025076	47.27306	46.2381	0.164832
Q791V5	Mtch2	0.301632559	0.000628	26.8073	30.47861	0.002116
P00397	Mtco1	0.461516405	0.009742	18.65026	18.50517	0.093851
P00405	Mtco2	0.470094879	0.000207	28.96597	26.76915	0.149
P00158	Mt-Cyb	-0.71055515	0.316188	24.97494	36.18323	0.18056

**Q9WTJ6**	**Mtl5**	**-0.49133588**	**0.325158**	**103.36195**	**73.03079**	**0.045373**
**P03888**	**Mtnd1**	**-3.0829241**	**0.295793**			
**P03911**	**Mtnd4**	**0.080030352**	**0.441215**			
**P03921**	**Mtnd5**	**0.430433822**	**0.019789**	**25.43842**	**22.19728**	**5.07E-05**
**O88441**	**Mtx2**	**0.897404962**	**0.165121**			
**Q5XKE0**	**Mybpc2**	**-0.715916**	**2.60E-16**	**17.05954**	**17.40371**	**0.001691**
**P70402**	**Mybph**	**-0.34945777**	**0.186054**	**37.16141**	**46.59007**	**0.036819**
**Q5SX40**	**Myh1**	**0.020937452**	**0.413057**	**37.31534**	**33.65629**	**0.033392**
**B1AR69**	**Myh13**	**-0.63680412**	**0.071043**	**44.41049**	**40.32745**	**0.213292**
**P13541**	**Myh3**	**0.262508232**	**0.377013**	**43.86551**	**18.45102**	**0.249615**
**Q5SX39**	**Myh4**	**-0.62994368**	**3.52E-20**	**32.76806**	**30.80558**	**3.13E-07**
**Q02566**	**Myh6**	**-0.61073343**	**0.23317**			
**Q91Z83**	**Myh7**	**-0.66717442**	**0.02275**	**29.22246**	**26.34069**	**0.016579**
**P13542**	**Myh8**	**-0.33597283**	**0.060198**	**47.2361**	**42.86863**	**0.115221**
**P05977**	**Myl1**	**-0.4541437**	**0.000247**	**58.7972**	**59.77678**	**0.029718**
**P51667**	**Myl2**	**0.070340653**	**0.402702**	**20.66503**	**15.8139**	**0.136358**
**P09542**	**Myl3**	**0.279440351**	**0.126659**	**12.80965**	**13.93551**	**0.011837**
**Q8VCR8**	**Mylk2**	**-2.09189286**	**0.02496**	**44.68053**	**72.44619**	**0.047492**
**P97457**	**Mylpf**	**-0.27983097**	**0.070565**	**52.2513**	**53.65413**	**5.07E-05**
**Q9JMH9**	**Myo18a**	**0.147176781**	**0.441873**			
**Q62234**	**Myom1**	**-0.28036587**	**0.040016**	**29.85285**	**32.49928**	**0.000119**
**Q9JIF9**	**Myot**	**-0.08087002**	**0.42479**	**8.31646**	**8.40335**	**0.09656**
**Q9JK37**	**Myoz1**	**-0.58725619**	**0.015114**	**22.77908**	**20.46918**	**0.071454**
**Q8R4E4**	**Myoz3**	**-0.14337049**	**0.392144**	**11.80449**	**22.28526**	**0.002116**
**Q8C5U4**	**NA**	**-2.52171634**	**0.268993**	**8.95091**	**24.20652**	**0.074867**
**Q8BHE8**	**NA**	**-1.02667983**	**0.316698**	**6.39803**	**10.66435**	**0.078237**
**Q99LU8**	**NA**	**NA**	**NA**	**7.91594**	**27.07707**	**0.176833**

**Q8C339**	**NA**	**-0.48552039**	**0.114469**	**18.28223**	**13.72695**	**0.199292**
**P06329**	**NA**	**-1.0958379**	**0.010797**			
**Q8K2T4**	**NA**	**0.827777795**	**NA**			
**Q9QUK4**	**Naip2**	**-0.21950507**	**0.325158**	**19.45898**	**23.75861**	**0.00434**
**Q6GQX2**	**Nckap5l**	**-0.58188082**	**0.195248**	**23.02692**	**33.82167**	**0.031098**
**O09000**	**Ncoa3**	**0.555375323**	**0.182522**	**35.80662**	**24.9924**	**0.098705**
**Q9WU42**	**Ncor2**	**-0.86008352**	**0.170128**	**1.92463**	**9.44916**	**0.000611**
**Q9QYG0**	**Ndrg2**	**-0.9257546**	**0.006233**	**2.91561**	**4.57996**	**0.162067**
**O35683**	**Ndufa1**	**0.388500079**	**0.049875**	**32.29813**	**27.241**	**0.227699**
**Q99LC3**	**Ndufa10**	**0.340342803**	**0.00185**	**26.68109**	**25.867**	**0.001577**
**Q9D8B4**	**Ndufa11**	**0.386251306**	**0.080069**	**22.00453**	**22.88779**	**0.054871**
**Q7TMF3**	**Ndufa12**	**0.518294391**	**0.059576**	**18.40511**	**17.63694**	**0.066496**
**Q9ERS2**	**Ndufa13**	**0.409061259**	**0.02496**	**23.44089**	**19.41382**	**0.151624**
**Q9CQ75**	**Ndufa2**	**0.568429623**	**0.071688**	**27.50922**	**29.42165**	**0.089449**
**Q9CQ91**	**Ndufa3**	**0.51130977**	**0.040713**	**21.00021**	**19.76669**	**0.049385**
**Q62425**	**Ndufa4**	**0.258724124**	**0.068764**	**13.81878**	**13.03819**	**0.256866**
**Q9CPP6**	**Ndufa5**	**0.485330724**	**0.028607**	**25.28198**	**26.65287**	**0.008745**
**Q9CQZ5**	**Ndufa6**	**0.560783923**	**0.01542**	**15.84313**	**15.35727**	**0.029718**
**Q9Z1P6**	**Ndufa7**	**-0.03105488**	**0.441354**	**12.12575**	**14.12613**	**0.003017**
**Q9DCJ5**	**Ndufa8**	**0.43113205**	**2.23E-09**	**33.9086**	**31.01781**	**0.016579**
**Q9DC69**	**Ndufa9**	**0.051263734**	**0.379563**	**23.85076**	**24.63906**	**0.00877**
**Q9CR21**	**Ndufab1**	**0.598593137**	**0.018876**	**41.91038**	**33.36359**	**0.213292**
**Q9DCS9**	**Ndufb10**	**0.317943472**	**0.000433**	**29.65731**	**28.04396**	**0.001657**
**O09111**	**Ndufb11**	**0.7841018**	**0.00519**	**24.94925**	**18.15107**	**0.174645**
**Q9CPU2**	**Ndufb2**	**0.279158124**	**0.16065**	**20.62173**	**18.15574**	**0.071883**
**Q9CQZ6**	**Ndufb3**	**0.428663606**	**0.00129**	**29.9716**	**30.4589**	**0.093851**
**Q9CQC7**	**Ndufb4**	**0.447972298**	**0.01161**	**25.23159**	**23.48679**	**0.032283**

**Q9CQH3**	**Ndufb5**	**0.416741871**	**0.001515**	**30.93564**	**32.93646**	**0.142265**
**Q3UIU2**	**Ndufb6**	**0.513747877**	**0.04204**	**13.27428**	**3.36314**	**0.237063**
**Q9CR61**	**Ndufb7**	**0.514360042**	**8.70E-05**	**38.12375**	**37.70128**	**0.020255**
**Q9D6J5**	**Ndufb8**	**0.341789226**	**0.061653**	**34.34346**	**30.35505**	**0.131738**
**Q9CQJ8**	**Ndufb9**	**0.346093377**	**0.04222**	**33.3288**	**27.02252**	**0.114912**
**Q9CQ54**	**Ndufc2**	**0.358666765**	**0.005086**	**21.03532**	**18.32418**	**0.029254**
**Q91VD9**	**Ndufs1**	**0.354746926**	**2.46E-06**	**20.1486**	**20.57246**	**0.00406**
**Q91WD5**	**Ndufs2**	**0.324544855**	**0.012022**	**15.41848**	**15.11706**	**0.011837**
**Q9DCT2**	**Ndufs3**	**0.363530184**	**0.014523**	**19.08552**	**18.24347**	**0.026176**
**Q9CXZ1**	**Ndufs4**	**0.582213156**	**0.007604**	**17.38605**	**15.9014**	**0.078292**
**Q99LY9**	**Ndufs5**	**0.417659107**	**0.035989**	**27.07505**	**26.90405**	**0.020255**
**P52503**	**Ndufs6**	**0.559949343**	**7.72E-08**	**22.0896**	**20.84079**	**0.020255**
**Q9DC70**	**Ndufs7**	**0.354072433**	**0.146939**	**11.16485**	**10.44628**	**0.059668**
**Q8K3J1**	**Ndufs8**	**0.270316036**	**0.16065**	**24.78909**	**20.97656**	**0.02829**
**Q91YT0**	**Ndufv1**	**0.458517533**	**2.95E-16**	**17.3066**	**17.59706**	**0.00057**
**Q9D6J6**	**Ndufv2**	**0.483703151**	**3.76E-05**	**21.16207**	**18.78552**	**0.244024**
**Q8BK30**	**Ndufv3**	**0.092802991**	**0.396066**	**33.60503**	**32.2185**	**0.136358**
**A2AQB2**	**Neb**	**-0.6742271**	**0.070565**	**43.72513**	**45.79133**	**0.029787**
**P08551**	**Nefl**	**-1.93582605**	**0.026313**	**19.75018**	**29.98681**	**0.047492**
**P08553**	**Nefm**	**-0.54995056**	**0.202104**	**54.61083**	**44.34206**	**0.048706**
**Q9Z1J3**	**Nfs1**	**-0.46237935**	**0.332388**	**6.26176**	**2.77242**	**0.122463**
**Q01768**	**Nme2**	**-0.46339913**	**0.159144**	**28.17581**	**19.6869**	**0.189727**
**Q9JKN6**	**Nova1**	**-0.58212592**	**0.413057**			
**Q60641**	**Nr1h4**	**-0.2935551**	**0.419058**			
**Q9DCN1**	**Nudt12**	**0.042759892**	**0.432634**	**24.27355**	**22.25728**	**0.029065**
**Q9DD16**	**Nudt22**	**-1.13180837**	**0.007665**	**39.73944**	**23.40991**	**0.098531**
**A2AAJ9**	**Obscn**	**-1.39198811**	**0.053827**	**12.93647**	**10.86463**	**0.166999**

**Q8CD62**	**Odz4**	**1.181620729**	**0.131169**	**270.21482**	**81.3615**	**0.094763**
**Q60626**	**Ofa**	**0.88545884**	**0.337874**			
**Q60597**	**Ogdh**	**0.419643447**	**1.89E-06**	**12.55652**	**12.56665**	**0.000327**
**P58281**	**Opa1**	**-0.22297685**	**0.37585**	**5.53489**	**2.68783**	**0.114912**
**Q8CIV2**	**ORF61**	**0.908077447**	**0.02823**	**5.28007**	**7.7091**	**0.143586**
**Q9D0K2**	**Oxct1**	**0.425816563**	**0.266617**			
**P09103**	**P4hb**	**0.05366942**	**0.358238**	**19.50717**	**20.87569**	**1.01E-06**
**Q99JB8**	**Pacsin3**	**-0.27051747**	**0.161788**	**4.76335**	**8.76104**	**0.020182**
**Q08642**	**Padi2**	**-1.11920433**	**0.003637**	**7.2626**	**7.86548**	**0.060199**
**Q640Q5**	**Pan3**	**-1.65470036**	**0.055278**	**120.50779**	**56.39391**	**0.086709**
**O88622**	**Parg**	**-0.32784671**	**0.27652**	**115.16185**	**92.00348**	**0.105252**
**Q99LX0**	**Park7**	**-1.65282615**	**0.000117**	**65.4099**	**71.93644**	**0.131738**
**Q3TVI8**	**Pbxip1**	**0.056125793**	**0.41667**	**11.75216**	**11.7098**	**0.000448**
**Q05920**	**Pc**	**0.219123303**	**0.269757**	**12.81202**	**14.56953**	**0.001271**
**P60335**	**Pcbp1**	**-1.40093434**	**0.251974**	**288.99057**	**217.50858**	**0.041601**
**Q99MN9**	**Pccb**	**0.927823679**	**0.001008**	**31.01061**	**42.77783**	**0.241516**
**Q9QYX7**	**Pclo**	**-0.61113156**	**0.266617**	**6.09094**	**10.03678**	**0.251095**
**P48725**	**Pcnt**	**-0.48168229**	**0.131169**	**28.27286**	**29.63346**	**0.001394**
**Q5DU28**	**Pcnxl2**	**0.084708378**	**0.431665**			
**Q922S4**	**Pde2a**	**-0.60055401**	**0.325158**	**17.47386**	**23.83041**	**0.229545**
**Q8CG03**	**Pde5a**	**0.384129364**	**0.313346**	**14.36487**	**5.09286**	**0.149524**
**P35486**	**Pdha1**	**0.75151494**	**4.43E-30**	**23.13575**	**25.92981**	**9.17E-09**
**P35487**	**Pdha2**	**-0.11881179**	**0.391456**	**18.16754**	**16.68422**	**0.143586**
**Q9D051**	**Pdhb**	**0.670527904**	**1.77E-08**	**58.24026**	**56.87072**	**0.03778**
**Q8BKZ9**	**Pdhx**	**0.617831579**	**2.62E-05**	**51.28325**	**43.75326**	**0.224306**
**Q8CI51**	**Pdlim5**	**-0.09313673**	**0.37585**	**34.82577**	**33.02916**	**0.003243**
**Q3TJD7**	**Pdlim7**	**0.052864683**	**0.428553**	**62.18129**	**52.42761**	**0.088748**

**P70296**	**Pebp1**	**-1.4757015**	**7.88E-07**	**122.6072**	**101.73405**	**0.031098**
**P47857**	**Pfkm**	**-0.52577897**	**1.29E-06**	**9.17933**	**9.29441**	**0.008745**
**O70250**	**Pgam2**	**-1.50141142**	**1.99E-11**	**34.23221**	**55.17365**	**9.38E-05**
**P09411**	**Pgk1**	**-1.15864756**	**3.14E-11**	**40.83287**	**39.61606**	**0.016469**
**Q9D0F9**	**Pgm1**	**-0.95167053**	**8.52E-06**	**41.07856**	**43.41773**	**0.001394**
**P67778**	**Phb**	**0.629203706**	**1.13E-13**	**28.34829**	**28.53352**	**0.000921**
**O35129**	**Phb2**	**0.668307645**	**0.001447**	**36.50837**	**40.00304**	**0.039864**
**Q9DAK9**	**Phpt1**	**-1.10373225**	**0.001607**	**3.78294**	**2.71**	**0.158669**
**Q8K4R4**	**Pitpnc1**	**-1.31915253**	**0.180185**			
**P52480**	**Pkm**	**-0.98985831**	**8.81E-26**	**25.98149**	**27.07543**	**3.03E-05**
**P27612**	**Plaa**	**0.16760619**	**0.294979**			
**P97813**	**Pld2**	**-0.34648672**	**0.392341**	**2.9287**	**2.47828**	**0.050453**
**Q9QXS1**	**Plec**	**-0.25553326**	**0.292843**	**25.97242**	**19.29538**	**0.174243**
**O88492**	**Plin4**	**0.191145376**	**0.300486**	**9.98626**	**13.21655**	**5.56E-05**
**J3QM92**	**Plscr5**	**0.175312924**	**0.350614**	**2.70841**	**1.4574**	**0.151476**
**Q9DAC9**	**Pou5f2**	**0.014110492**	**0.453678**	**0.82251**	**1.61692**	**0.035998**
**Q3UM45**	**Ppp1r7**	**0.011940797**	**0.458227**	**31.399**	**43.33345**	**0.020191**
**Q61171**	**Prdx2**	**-0.6921004**	**0.120383**	**29.59044**	**27.98804**	**0.07013**
**P20108**	**Prdx3**	**0.496801765**	**0.006959**	**18.36213**	**16.97419**	**0.008139**
**P99029**	**Prdx5**	**0.228404165**	**0.157751**	**23.78312**	**20.83743**	**0.000105**
**Q9QUN5**	**Prl3c1**	**-0.14398782**	**0.429246**	**30.79479**	**29.09106**	**0.177348**
**O55103**	**Prx**	**-0.10396203**	**0.392144**	**73.12179**	**55.57249**	**0.005951**
**Q61207**	**Psap**	**-0.23766942**	**0.20234**	**3.92565**	**3.90139**	**0.034494**
**Q9JM51**	**Ptges**	**-0.92520282**	**0.222622**	**28.8113**	**11.22158**	**0.195781**
**Q9QY80**	**Ptpla**	**-0.39493161**	**0.033352**	**346.53517**	**196.94927**	**0.14864**
**Q62130**	**Ptpn14**	**-0.22879176**	**0.244459**	**6.69652**	**8.0295**	**0.208378**
**P32848**	**Pvalb**	**-1.53841263**	**1.81E-20**	**61.15077**	**65.88911**	**0.016746**

**Q9ET01**	**Pygl**	**-2.00939154**	**0.002815**	**37.20376**	**34.2902**	**0.237341**
**Q9WUB3**	**Pygm**	**-1.03873529**	**6.20E-21**	**19.5713**	**18.01343**	**0.000123**
**P61027**	**Rab10**	**-0.12316574**	**0.313165**	**7.75058**	**6.73858**	**0.114912**
**O35963**	**Rab33b**	**0.222185367**	**0.121582**	**6.03754**	**5.65076**	**0.119491**
**A2AWA9**	**Rabgap1**	**1.019331617**	**0.122275**	**1.27479**	**0.6802**	**0.020255**
**P63001**	**Rac1**	**0.094300013**	**0.423938**	**10.89843**	**12.45521**	**0.015809**
**E9Q428**	**Rad51l3**	**0.44351818**	**0.392965**			
**Q75NR7**	**Recql4**	**-0.97641739**	**0.101797**	**19.40238**	**23.62706**	**0.093851**
**Q9R1A8**	**Rfwd2**	**-1.19395118**	**0.301635**	**7.49049**	**7.60055**	**0.095969**
**Q9CQE5**	**Rgs10**	**1.261385428**	**0.023088**	**9.90315**	**16.82732**	**0.267132**
**Q9CZM2**	**Rpl15**	**-0.0692518**	**0.428822**	**10.35469**	**10.55323**	**0.151476**
**P62889**	**Rpl30**	**0.649991353**	**0.199197**	**6.311**	**10.04448**	**0.006233**
**P14869**	**Rplp0**	**1.60736136**	**0.041972**	**11.70546**	**7.55645**	**0.100404**
**Q91YQ5**	**Rpn1**	**-0.18895443**	**0.27652**	**25.608**	**46.32408**	**0.020106**
**Q9WUT3**	**Rps6ka2**	**-0.45576615**	**0.266617**	**5.19094**	**8.74066**	**0.02684**
**Q8CBB9**	**Rsad2**	**0.947161164**	**0.047466**			
**Q7M732**	**Rtl1**	**-0.62466149**	**0.27652**			
**O70622**	**Rtn2**	**-0.38862979**	**0.001447**	**29.72495**	**31.00477**	**0.102775**
**E9PZQ0**	**Ryr1**	**-0.20433814**	**5.91E-06**	**21.96747**	**21.37986**	**4.95E-23**
**Q8BGH2**	**Samm50**	**1.008719311**	**3.92E-05**	**27.65529**	**28.5059**	**0.003765**
**Q8K2B3**	**Sdha**	**0.603670098**	**1.38E-39**	**15.74486**	**14.88739**	**0.002005**
**Q9CQA3**	**Sdhb**	**0.355439511**	**0.021688**	**15.07007**	**13.23361**	**0.026991**
**Q9CXV1**	**Sdhd**	**0.373538353**	**0.194652**	**87.06494**	**80.69671**	**0.135532**
**Q8R0F9**	**Sec14l4**	**0.591373834**	**0.201641**	**56.5797**	**14.88448**	**0.135271**
**P70274**	**Sepp1**	**-0.38742597**	**0.18945**	**6.33612**	**5.84082**	**0.031163**
**O70456**	**Sfn**	**-0.05032925**	**0.437693**	**196.98764**	**159.19611**	**0.186929**
**P82348**	**Sgcg**	**1.112640764**	**0.063661**	**22.14079**	**16.01383**	**0.149524**

Q62141	Sin3b	-0.26313349	0.391456	64.45292	39.74152	0.12838
P46062	Sipa1	-0.12158694	0.397862			
Q9CR62	Slc25a11	-1.25937447	0.073305	7.24855	4.55868	0.2157
Q8BH59	Slc25a12	0.286511826	8.52E-06	27.9801	26.84628	0.001394
Q9QXX4	Slc25a13	-0.33255199	0.325158	48.09643	42.26728	0.002005
Q9Z2Z6	Slc25a20	-0.87079456	0.159144	25.45335	26.97975	0.117498
Q8VEM8	Slc25a3	0.403209279	2.30E-05	24.12496	23.44273	0.011794
P48962	Slc25a4	0.215571297	0.190245	36.81409	35.04731	0.054871
P51881	Slc25a5	0.32629549	0.257618	31.22353	28.57031	0.07839
P14142	Slc2a4	-1.32628946	0.007374			
Q8BYF6	Slc5a8	-1.18501628	0.150138			
Q9D8T7	Slirp	-0.55167735	0.329619	8.03733	6.43609	0.176153
Q8C1Q6	SMIM4	-1.89461577	0.126946	15.35219	32.25127	0.112086
Q8BQA2	Snap91	-0.59957751	0.178562	81.29233	88.5439	0.042759
P08228	Sod1	-0.95995714	9.44E-05	31.82702	34.44256	0.047199
P09671	Sod2	0.425317206	0.02275	33.37978	20.85018	0.085454
Q62407	Speg	-0.18259552	0.349922	1.87245	4.57375	0.069347
Q3ULF4	Spg7	-1.48086605	0.03115			
Q8C804	Spice1	-1.55107962	0.14269	71.69063	186.82588	0.140141
Q91WK1	Spryd4	0.447314171	0.02287	13.56897	10.82757	0.131738
Q7TQ48	Srl	-0.13819262	0.199197	70.4547	63.84463	0.000279
Q9CYR0	Ssbp1	0.111927186	0.397801	27.44131	23.18016	0.001007
Q76K27	St6gal2	-0.41156369	0.37773	31.36744	18.22764	0.182088
Q8BZ71	Stac3	-0.91335804	4.98E-05	1.51268	1.56636	0.133926
Q9JMD3	Stard10	-0.01901538	0.449507	1.60423	1.97907	0.018755
Q8C7E7	Stbd1	-0.24785233	0.294979	7.14148	7.03275	0.163276
Q99JB2	Stoml2	-0.11710825	0.41559	9.75959	6.49388	0.253985

Q9Z2I9	Sucla2	0.187054578	0.226844	21.72167	20.24363	0.014493
Q9WUM5	Suclg1	0.683016792	0.018521	34.4207	31.45309	0.222511
Q8BJS4	Sun2	0.221080651	0.396055	15.76954	19.35838	0.117066
Q6ZWR6	Syne1	-0.39317914	0.241361	7.87877	5.39495	0.244169
O89104	Sypl2	-0.05122427	0.428822	97.17714	120.91441	0.091894
Q920N7	Syt12	-0.23312124	0.379563	2.66145	0.77744	0.227699
P40749	Syt4	0.438805886	0.182522	8.57661	5.54663	0.103009
E9Q8T1	Tacc2	0.343138206	0.392144			
Q7M724	Tas2r106	1.057074471	0.27652	74.29655	30.54283	0.157725
P70323	Tbx1	-0.14358143	0.36087	8.03549	9.75966	0.0099
P23881	Tcea3	4.495219229	0.293774			
P30051	Tead1	1.034557202	0.131169			
Q8CD34	Tead3	-0.28149095	0.37773	9.6388	8.19148	0.071526
Q9CY27	Tecr	-0.1524462	0.312424	30.35341	23.82208	0.249477
Q99MW5	Tex13	-0.23511525	0.388937	14.83925	12.70857	0.022999
Q921I1	Tf	0.202799109	0.380286	1.90097	3.02064	0.044062
Q923W1	Tgs1	0.058880605	0.428553	3.73469	4.87259	0.047135
P63058	Thra	0.091493521	0.392144	6.08512	6.98404	0.018071
Q69ZU6	Thsd7a	-0.2723718	0.294979	10.92305	8.40937	0.155759
P62075	Timm13	0.57349054	0.097523	4.53587	4.0125	0.232684
Q9D880	Timm50	-0.99095202	0.18945	37.74547	57.87956	0.045373
Q3UBX0	Tmem109	1.844079166	0.301635			
Q8C2L6	Tmem161b	0.322646037	0.419058	42.77162	24.14486	0.199292
Q9CZ16	Tmem178a	-0.30575884	0.358238	51.4365	51.87567	0.010231
Q3TMP8	Tmem38a	-0.47962686	0.212424	30.06003	33.72971	0.02663
Q8C0L0	Tmx4	1.292974303	NA			
P20801	Tnnc2	-0.59507737	2.69E-05	24.79857	24.81704	0.047199

P13412	Tnni2	-0.9580672	0.000543	17.42599	19.66007	0.004598
Q9QZ47	Tnnt3	-0.45782256	0.001892	18.86305	18.17033	0.054452
P17751	Tpi1	-0.8939044	3.72E-11	34.35976	33.42765	0.000126
P58771	Tpm1	-0.59152861	0.000167	46.2364	42.99637	0.000448
P58774	Tpm2	-0.60641153	1.37E-08	56.24393	57.20447	0.001394
P21107	Tpm3	-0.00295684	0.462271	74.17313	70.08041	0.138439
Q6IRU2	Tpm4	-0.4096794	0.152858	24.53758	24.14709	0.044531
Q1XH17	Trim72	-0.24730694	0.021253	14.43944	14.8551	3.54E-06
Q9D0C4	Trmt5	-0.38881283	0.317798	7.65701	3.88433	0.15307
Q9Z2Q2	Tsg118	-0.27192118	0.388937	41.96263	28.98464	0.24496
A4Q9E4	Ttll2	-0.08899499	0.45269			
Q8BUJ0	Ttn	-0.39578606	0.032782	29.37602	30.90045	3.18E-79
A2ASS6	Ttn	-0.34739268	1.75E-27	22.05253	24.32479	6.30E-05
Q8BFR5	Tufm	0.698438579	9.41E-16	25.2091	23.09145	0.002923
Q6ZWZ2	Ube2r2	0.605569086	0.061126			
Q9ES34	Ube3b	0.24469369	0.390447	5.77463	2.04407	0.206785
A2AN08	Ubr4	0.548323326	0.318517			
Q6P5E4	Uggt1	-0.1606101	0.228676	12.87393	14.05475	0.007179
Q91ZJ5	Ugp2	-1.41104726	0.000628	26.50555	30.96311	0.051876
Q9D855	Uqcrb	0.699181514	1.89E-06	33.94769	36.96316	0.07839
Q9CZ13	Uqcrc1	0.410389379	4.26E-07	32.22307	31.4677	8.64E-05
Q9DB77	Uqcrc2	0.194347705	0.070565	34.83622	34.09747	0.02419
Q9CR68	Uqcrfs1	0.223685223	0.058406	23.11684	22.72725	0.000648
P99028	Uqcrh	0.926854139	0.002068	33.19157	34.70946	0.01087
Q9CQ69	Uqcrq	-0.31790301	0.161788	34.60589	38.54227	0.205343
Q78IK2	Usmg5	0.34273793	0.049244	25.25177	26.14329	0.031098
Q9QY76	Vapb	0.349074732	0.046555	40.87336	41.82608	0.016579

**Q01853**	**Vcp**	**0.723564724**	**4.14E-14**	**6.93557**	**7.63674**	**8.98E-07**
**Q60932**	**Vdac1**	**0.149170754**	**0.072721**	**29.44646**	**28.09688**	**0.182088**
**Q60930**	**Vdac2**	**0.152869954**	**0.159144**	**26.81229**	**24.87708**	**0.141271**
**Q60931**	**Vdac3**	**-0.0004835**	**0.462477**	**27.45624**	**25.22476**	**0.154065**
**Q9EQH3**	**Vps35**	**-2.41340471**	**0.047466**	**46.91092**	**26.98795**	**0.221709**
**Q8R0J7**	**Vps37b**	**0.102829279**	**0.380286**			
**Q4VBE8**	**Wdr18**	**1.179786983**	**0.031346**	**39.83876**	**31.95524**	**0.052472**
**Q8BND3**	**Wdr35**	**0.320024724**	**0.20234**	**7.65131**	**13.14212**	**0.000513**
**Q9R0D8**	**Wdr54**	**-0.50722839**	**0.24835**	**17.97489**	**37.06312**	**0.011565**
**Q9D565**	**Wdr64**	**0.787963398**	**0.421009**			
**Q6P5F9**	**Xpo1**	**0.081984018**	**0.422175**			
**P46938**	**Yap1**	**0.198948308**	**0.413057**	**17.02393**	**8.97462**	**0.202653**
**P62259**	**Ywhae**	**-0.51033092**	**0.001252**	**19.84762**	**22.42108**	**0.009832**
**P61982**	**Ywhag**	**0.73407257**	**0.121312**	**23.30617**	**15.82422**	**0.025986**
**Q69ZB8**	**Zcchc2**	**-0.44151452**	**0.309859**	**1400.23747**	**1287.62785**	**0.154251**
**P10755**	**Zfp14**	**-0.71380956**	**0.097555**	**7.34319**	**6.98841**	**0.092644**
**Q9JLM4**	**Zmym3**	**0.414027471**	**0.288499**	**10.85376**	**4.74341**	**0.163376**

SOL

		Abundance, YCL/OCL		Turnover, OCL	Turnover, YCL	
UniProtKB	Gene ID	log2 fold change	q value	t/2 (d)	t/2 (d)	q value
Q78IR3	4632415L05Rik	0.41969	0.5159	7.97301	4.37872	0.19381
E9Q1N0	4932431P20Rik	-0.04362	0.692918	1.52321	1.14136	0.293916
Q9JI39	Abcb10	0.33226	0.44784			
Q9DBL9	Abhd5	0.34529	0.687378	19.15564	22.15008	0.150731
Q8BWT1	Acaa2	0.27346	0.000445	24.53208	22.86126	2.86E-02
Q8JZN5	Acad9	0.13667	0.611601	13.64768	13.96896	0.028989

P51174	Acadl	0.38254	1.60E-06	24.08242	22.25679	0.002694
P45952	Acadm	0.07332	0.633434	14.63864	14.70568	0.18034
Q07417	Acads	0.45318	0.004546	27.81205	24.30808	0.349525
P50544	Acadvl	-0.07714	0.390619	17.22075	15.27691	2.98E-01
Q8BRH7	Acap2	-0.48321	0.668743			
Q8QZT1	Acat1	0.21354	0.007664	23.62417	20.90777	0.197385
Q99KI0	Aco2	0.28082	6.46E-05	26.94066	24.66584	0.065782
Q9CQR4	Acot13	0.44764	0.139747	31.46182	27.7861	0.244336
Q9QYR9	Acot2	-0.82838	0.143953	24.73252	23.4585	0.011701
Q9QXD1	Acox2	-1.26279	0.35604	12.82813	9.53939	0.289801
Q3UZN1	Acp2	-0.34284	0.430822	7.53097	5.04304	0.215166
P41216	Acsl1	-0.15021	0.089478	19.2766	18.49832	0.267116
P68134	Acta1	0.33516	0.367136	109.0098	97.81936	0.000676
Q9JI91	Actn2	-0.10815	0.479942	25.25028	24.66828	5.83E-03
O88990	Actn3	0.3597	0.022067	10.94545	9.15267	2.45E-04
Q60936	Adck3	0.3955	2.79E-05	4.02405	3.66412	1.21E-05
P54822	Adsl	-1.74103	0.121435	11.96551	18.42048	0.215851
P28650	Adssl1	-0.70952	0.045322	16.07641	14.45906	0.135365
Q8JZQ2	Afg3l2	3.57205	0.566131			
Q8N9S3	Ahsa2	-0.68745	0.442578	34.34913	35.78605	0.082587
P29699	Ahsg	-0.74363	0.454112	0.78296	0.37324	0.180737
Q9Z0X1	Aifm1	-0.00248	0.709759	33.79373	30.72074	3.63E-04
Q9R0Y5	Ak1	-0.60357	0.059572	11.3876	6.56862	0.223275
P45376	Akr1b1	-0.97766	0.000766	12.85716	12.23705	0.254752
P45377	Akr1b8	0.05872	0.668743	39.36632	34.1351	0.061077
Q9D1F4	Akt1s1	-0.66996	0.154119	2.34991	1.17952	0.082587
P07724	Alb	-0.79784	4.09E-15	1.48183	1.38663	1.21E-10

P47738	Aldh2	0.62122	0.001309	6.67975	6.13227	0.277024
Q8CHT0	Aldh4a1	0.47722	0.098715	22.73472	25.1421	0.359121
Q9EQ20	Aldh6a1	0.19487	0.098715	20.99318	19.32515	0.047524
P05064	Aldoa	-0.81405	3.07E-13	15.65952	14.66308	0.00751
Q3V1D3	Ampd1	-0.4087	0.57607	9.98199	7.72516	0.298175
Q02357	Ank1	-0.04192	0.686022	3.05554	12.83421	0.005775
G5E8K2	Ank3	-0.08159	0.668743	2.84086	5.55586	0.156788
Q505D1	Ankrd28	-0.55385	0.668743	1.47938	0.78659	0.324555
Q8K298	Anln	-0.11726	0.668743			
P07356	Anxa2	0.22046	0.229447	21.23493	17.02934	0.141981
P48036	Anxa5	0.43604	0.447476			
P14824	Anxa6	-0.63332	0.011057	15.3274	16.74904	0.051481
Q9D7N9	Apmap	2.58923	0.286449			
Q9WV35	Apobec2	0.09194	0.637095	14.58785	14.90104	0.090547
Q9DCZ4	Apoo	-0.06253	0.624835	27.70376	27.40313	0.035273
Q78IK4	Apool	0.02758	0.687158	45.68909	45.32856	0.352841
Q8BXL7	Arfrp1	1.18771	NA			
Q61210	Arhgef1	-2.70921	0.145745	1.12391	2.8043	0.152929
Q8R5J9	Arl6ip5	0.1033	0.670985	6.22306	5.85479	0.292088
Q8R2G4	Art3	0.06964	0.692918	20.8236	18.81415	0.02123
Q8BSY0	Asph	-0.32491	0.245149	68.76602	64.71066	0.349525
Q99MQ4	Aspn	0.18797	0.668743	528.78531	436.03674	0.269305
Q61137	Astn1	-0.01731	0.709759	3.87248	1.93276	0.160077
Q6PA06	Atl2	-0.27398	0.392282	3.94429	3.79278	0.11727
Q8VDN2	Atp1a1	-0.01043	0.700856	10.72332	8.80328	0.237903
Q6PIE5	Atp1a2	-0.02083	0.668743	5.3685	4.67136	2.08E-01
P14094	Atp1b1	-0.40678	0.260674	7.60334	8.3953	0.185692

P14231	Atp1b2	0.06823	0.674733	4.52072	1.38616	0.314248
Q8R429	Atp2a1	-0.38048	0.004029	15.90926	15.36551	0.026502
F6RQN3	Atp2a1	-0.24074	0.430822	11.20332	12.96649	0.156935
Q64518	Atp2a3	-0.24931	0.297889	10807.3492	11244.80292	0.069887
Q03265	Atp5a1	0.0091	0.687158	23.61646	20.96757	0.104175
P56480	Atp5b	0.17432	0.016069	25.58428	23.37698	0.343003
Q91VR2	Atp5c1	-0.149	0.236894	31.24353	30.25072	0.184155
Q9D3D9	Atp5d	0.22807	0.157359	36.36504	34.54863	0.180272
P56382	Atp5e	0.10968	0.502217	30.61849	27.60368	0.051294
Q9CQQ7	Atp5f1	-0.35647	0.275173	30.29023	27.90482	0.171911
Q9DCX2	Atp5h	-0.19188	0.068541	43.53926	39.59681	0.005784
Q06185	Atp5i	0.04553	0.674733	29.98733	28.74046	0.182616
P97450	Atp5j	-0.14427	0.562517	26.00379	22.82613	0.06542
Q9DB20	Atp5o	-0.21499	0.259395	38.46544	34.84679	0.194745
A3FIN4	Atp8b5	-0.34184	NA			
Q8R087	B4galt7	-2.15485	0.342238	4.43482	5.21642	0.089801
Q8CGM1	Bai2	0.09238	0.674733	9.31639	7.7209	0.237712
Q8R1X0	BC022960	-1.13305	0.370707	30.69516	30.70657	0.268374
Q61335	Bcap31	-0.91378	0.218467	13.7938	15.96592	0.015642
P59017	Bcl2l13	0.02718	0.670985	21.77776	20.316	0.107284
Q80XN0	Bdh1	-0.0761	0.668743	36.24389	28.6307	0.000397
O08539	Bin1	0.04225	0.668743	9.8339	10.28499	0.005827
O55003	Bnip3	0.1066	0.656106	1.67757	3.78386	0.127575
P18572	Bsg	-0.32426	0.245149	10.1881	8.60591	0.090153
Q8CFE5	Btbd7	0.13314	0.674733			
Q7TNF8	Bzrap1	0.76334	0.492804			
Q8R066	C1qtnf4	-0.01754	0.709759	51.57654	23.76356	0.229786

Q8CG14	C1sa	0.33188	0.443304	27.53799	13.78975	0.136339
P16015	Ca3	-0.98329	2.15E-16	28.74712	34.02264	3.37E-06
Q02789	Cacna1s	0.09432	0.665254	15.8776	12.48263	0.060217
O08532	Cacna2d1	-0.23176	0.168622	22.45097	21.07201	0.055747
Q8R3Z5	Cacnb1	0.03633	0.674733	15.57793	17.98691	0.105266
Q3UKW2	Calm1	-0.01312	0.668743	14.69485	25.13356	0.038449
P62204	Calm1	0.20263	0.697646	16.62198	14.00587	0.183903
P14211	Calr	-0.28288	0.491706	8.01825	8.47698	0.00221
O35887	Calu	-0.3756	0.275173	10.88159	10.9678	0.310997
P11798	Camk2a	-0.02333	0.700458	4.27333	7.71385	0.055958
O09165	Casq1	-0.14252	0.479942	57.87585	58.1664	5.83E-03
O09161	Casq2	-0.30381	0.267362	54.01516	54.24017	0.017005
P49817	Cav1	-0.10936	0.594311	13.49717	14.18047	0.021916
Q9DA08	Ccdc101	-0.24274	0.547663	6.01025	5.56085	0.016911
Q3TMW1	Ccdc102a	-0.17427	0.668743	15.11478	15.85495	0.109633
Q3UX62	Ccdc114	0.29145	0.674733	19.43901	61.00198	0.026239
Q810T2	Ccnb3	-0.12474	0.668743	14.26515	16.97963	0.005775
Q08857	Cd36	0.19393	0.479942	6.34178	5.85835	0.060217
Q7TT50	Cdc42bpb	0.27578	0.510367	9.725	6.23987	0.289069
Q6A068	Cdc5l	-0.97971	0.172811			
Q9WTR5	Cdh13	0.1523	0.503714	11.36581	15.46755	0.036723
Q91X79	Cela1	-0.28506	0.245149	22.82669	21.3098	0.323994
Q9JKC6	Cend1	-1.6595	NA			
Q6A065	Cep170	-0.45398	0.373903	245.49176	152.53799	0.152626
Q3UPP8	Cep63	-4.04122	NA			
Q8VCT4	Ces1d	0.35659	0.668743	3.41742	4.18363	0.135077
P45591	Cfl2	-1.16945	0.020609	35.39327	23.20855	0.324555

Q6AW69	Cgnl1	-1.00829	0.318322	24.12064	42.4324	0.205129
Q9CRB9	Chchd3	-0.19557	0.157359	32.12837	27.8094	2.68E-02
P26339	Chga	-0.10318	0.670985	11.40225	11.33366	0.145738
Q8BIW9	Chtf18	0.10243	0.700458			
Q91WS0	Cisd1	0.13376	0.506089	25.33626	24.54293	0.329179
B1AR13	Cisd3	0.38347	0.245149	11.52139	1.55482	0.051076
P07310	Ckm	-1.44575	1.16E-38	20.55762	22.55548	1.21E-05
Q6P8J7	Ckmt2	-0.1201	0.370043	27.25987	24.26307	0.132534
Q68FD5	Cltc	-0.32821	0.499827	5.97613	6.0132	0.138777
Q8R4N0	Clybl	0.03061	0.687158	59.84319	60.99552	0.030227
Q8BH15	Cnot10	-0.32889	0.60832	3.09892	7.51639	0.280064
P16330	Cnp	1.05799	0.433967	47.18761	10.21173	0.106483
P11087	Col1a1	-0.11093	0.670985	91.83001	58.21502	0.349525
Q01149	Col1a2	0.18081	0.637095	2.68918	1.84582	0.361837
P28481	Col2a1	-0.36483	0.135328	13.16725	13.30783	0.00441
Q8BMS4	Coq3	0.46162	0.019501	21.89826	32.55184	0.177924
Q8K1Z0	Coq9	0.55211	8.90E-05	18.52381	16.45069	0.326292
P19783	Cox4i1	-0.0551	0.659117	27.43344	25.35953	0.051816
P12787	Cox5a	-0.43823	0.000113	22.24527	20.98042	0.051816
P19536	Cox5b	-0.21269	0.275173	25.40857	22.15046	0.229786
P43024	Cox6a1	-0.05659	0.674733	14.63903	13.05986	0.212994
P43023	Cox6a2	-0.32381	0.388185	8.48943	7.64934	0.155851
P56391	Cox6b1	-0.30305	0.414697	28.50196	27.78296	0.114639
Q9CPQ1	Cox6c	0.01069	0.709759	25.48069	22.18448	0.003628
P56392	Cox7a1	0.04671	0.670985	18.85503	16.92039	0.298246
P48771	Cox7a2	0.04673	0.668743	10.1222	9.47309	0.182448
Q0VE82	Cpne7	1.82401	0.154231			

Q924X2	Cpt1b	-0.10027	0.377008	14.95431	13.48353	0.055747
P52825	Cpt2	0.13325	0.327978	20.02132	17.97553	0.026502
P47934	Crat	0.64652	1.39E-06	25.65074	22.07657	0.040356
Q9D2A5	Creb3l4	0.2309	0.668743	1229.62307	316.5755	0.323994
P23927	Cryab	-1.0067	1.58E-06	8.34604	8.55446	0.006055
Q9CZU6	Cs	0.52313	6.59E-07	26.66626	24.60284	0.310892
Q99388	Csprs	0.56922	0.502217			
P18242	Ctsd	0.2139	0.296886	6.61327	3.84003	0.037406
Q9CQX2	Cyb5b	0.22893	0.5159	13.46733	11.57763	0.016379
Q9DB73	Cyb5r1	-0.04586	0.700458	29.30039	21.55312	0.311985
Q9D0M3	Cyc1	-0.12908	0.388185	33.85089	31.66101	0.305918
P62897	Cycs	0.26152	0.060313	29.59356	27.2921	0.060217
B7ZWD6	Cym	-0.62863	NA			
Q9QX11	Cyth1	-0.68038	0.174198	13.99546	36.51975	0.000571
Q9D172	D10Jhu81e	0.36417	0.01749	23.97999	23.77883	0.152929
P31786	Dbi	-0.6315	0.078819	22.12852	25.11588	0.036396
Q8BGW4	Dcaf12l2	0.53232	0.199996	17.45412	14.81155	0.29947
Q9JLM8	Dclk1	0.32139	0.542775	21.73032	20.78059	0.059204
P28654	Dcn	-0.27405	0.325187	6.29608	5.73757	0.104671
Q9CQ62	Decr1	0.53695	7.52E-10	24.87157	23.59299	0.000167
P31001	Des	-0.04325	0.668743	9.60299	9.10108	0.002837
Q99LB2	Dhrs4	-0.01346	0.709759	31.34081	33.02107	0.180422
Q8CHS7	Dhrs7c	-0.13188	0.388185	13.82171	15.42084	0.007522
Q8BMF4	Dlat	0.30191	6.94E-05	39.25565	36.75673	0.127575
O08749	Dld	0.36036	3.86E-05	34.5867	30.70934	0.014782
Q9D2G2	Dlst	0.14891	0.373903	33.84758	32.55916	0.150731
Q8VHE6	Dnah5	1.35635	0.325121	9.08251	5.09566	0.359878

Q9QYI6	Dnajb9	-0.62202	0.089819	7.39144	5.46114	0.034341
D3Z2X2	Dnhd1	-0.44523	0.388185			
A2AF47	Dock11	-0.0596	0.633434			
O08553	Dpysl2	0.7977	0.345007	6.73874	2.97488	0.000117
Q8BLI4	Dse	-0.13903	0.568382	4.64407	4.06481	0.012569
Q9D2N4	Dtna	1.05646	0.067035	14.53213	11.65533	0.215166
O35459	Ech1	0.63486	0.149044	4.20201	3.77317	0.023383
Q8BH95	Echs1	0.12118	0.454112	31.55367	29.22405	0.047043
P42125	Eci1	-0.01862	0.700139	24.97821	22.99124	0.031051
Q9WUR2	Eci2	-0.01605	0.708326	14.6249	9.70001	0.180422
P62631	Eef1a2	-0.06193	0.665254	14.106	13.39318	0.180489
P58252	Eef2	-0.56032	0.020182	7.28918	8.30732	0.003013
Q69ZW3	Ehbp1	2.15359	0.559611			
Q8BH64	Ehd2	0.36221	0.07365	16.55207	11.53481	0.080502
Q99LC8	Eif2b1	1.88411	0.245149			
P17182	Eno1	0.25841	0.547558	16.44409	17.72079	0.024621
P17183	Eno2	1.67188	0.068541	6.69059	6.37456	0.088676
P21550	Eno3	-0.63983	3.33E-05	21.04878	22.99934	0.00751
O70318	Epb41l2	0.59351	0.44784	18.98721	8.79339	0.307962
Q99M71	Epdr1	-0.29808	0.089076	5.37481	5.90458	0.031051
Q99LC5	Etfa	0.10183	0.393605	25.85279	23.37246	1.55E-01
Q9DCW4	Etfb	0.21392	0.106677	25.14121	23.5201	0.079401
Q921G7	Etfdh	-0.16618	0.134333	27.13726	25.8909	0.310997
Q9DCM0	Ethe1	0.36757	0.661174	23.35067	17.3015	0.090153
Q8BI71	Exoc3l1	0.91172	0.603511	76.80855	33.07537	0.150131
Q8R313	Exoc6	-0.45822	0.477847			
P55050	Fabp2	0.44559	0.553469	20.74726	14.6671	0.158749

P11404	Fabp3	-1.54515	3.58E-08	27.3191	25.64874	0.024664
P04117	Fabp4	-0.27002	0.367937	13.49706	11.20104	0.056485
O08716	Fabp9	-0.36585	0.589517	8.48501	5.87712	0.189946
Q8R0F8	Fahd1	-0.04827	0.690701	22.30209	22.54527	0.116788
Q3U2I3	Fam160a2	-1.76474	0.134333			
Q9D6U8	Fam162a	0.33439	0.420405	14.66607	12.70435	0.000167
Q14CH0	Fam171b	-1.16025	0.496361	9.08363	19.94127	0.073753
Q61554	Fbn1	0.96832	0.089076	598.323	167.3833	0.091683
Q78JE5	Fbxo22	-0.38055	0.632945			
Q8BI58	Fbxw26	0.01604	0.711313	3.06986	9.06295	0.033468
P70451	Fer	-0.07584	0.674733	8.64353	22.1013	0.059204
P97807	Fh	0.28286	0.004362	28.37272	26.71661	6.97E-04
P97447	Fhl1	-0.76763	0.006787	20.00988	20.80368	0.348822
Q91V79	Fitm1	-0.59451	0.019501	2.95317	2.58143	0.003628
Q8VHX6	Flnc	-0.00355	0.711313	7.18838	8.24152	0.001033
Q8R2I0	Foxe1	4.20784	0.001518	16.83164	18.00053	0.004064
E9Q8I9	Fry	0.50377	0.377905			
Q32M02	Fscn2	-0.21741	0.668743	19.51139	12.77582	0.28327
P16858	Gapdh	-0.86184	4.27E-07	20.44881	23.45419	0.078577
P17679	Gata1	-1.85525	0.198146	1.33008	1.27255	0.197983
O55126	Gbas	0.00711	0.709759	26.9466	25.1104	0.007522
Q5SV77	Ggnbp2	-2.13728	0.287206			
Q80Y14	Glrx5	-0.32435	0.603511	9.80444	8.46267	0.269305
P26443	Glud1	0.3074	0.260674	22.91325	23.896	0.248699
Q3UN64	Gm10564	0.99666	0.420405			
Q3TNV0	Gm10941	-1.53904	0.479942	6.59769	6.42367	0.164023
D3Z1B2	Gm4953	-0.19334	0.418914	11.76685	4.46921	0.348822

Q3ULJ8	Gm4981	0.76259	0.245149			
D3Z1Y8	Gm6483	1.09082	0.154231	7.94225	8.51148	0.110922
F6QVC4	Gm9732	0.63517	0.5364	40.68544	29.2844	0.267116
Q9DCZ1	Gmpr	-0.45344	0.388185			
Q6PGG6	Gnl3l	1.7436	0.532642			
Q692V3	Gnn	-0.73161	0.134333	39.58422	44.01787	0.093889
P05201	Got1	-1.20268	2.88E-15	17.67203	21.72766	0.002599
P05202	Got2	0.34587	3.73E-05	29.84075	26.85575	0.344861
P13707	Gpd1	-0.00958	0.709759	5.76735	5.53613	0.200743
Q64521	Gpd2	-0.01082	0.692918	13.25104	14.14605	7.79E-05
P06745	Gpi	-0.58911	0.002123	10.10505	16.79201	1.06E-02
Q8VEC3	Gpr110	2.51649	0.260076			
Q8C010	Gpr61	0.42267	0.637095	26.80587	15.42186	0.244336
Q03391	Grin2d	0.26249	0.668743	9.3705	5.40548	0.272637
P10649	Gstm1	-1.35749	0.007058	3.99604	20.06419	0.203334
P52785	Gucy2e	-0.03652	0.70504	5.454	7.89975	0.237712
Q9Z1E4	Gys1	-0.99189	0.083975	6.72583	6.57618	0.18946
Q31168	H2-D1	-0.46669	0.610951			
Q61425	Hadh	0.10454	0.492804	24.12784	22.41934	0.001996
Q8BMS1	Hadha	-0.04217	0.510407	26.94161	24.88674	3.02E-04
Q99JY0	Hadhb	0.08165	0.388185	14.84048	13.41222	0.140905
P01942	Hba	-1.68537	0.008237	30.70238	37.33807	0.15545
P02088	Hbb-b1	-1.17383	0.007912	28.1597	35.78549	0.007549
Q7TQ32	Hfe2	0.66742	0.465893	45.43906	45.70593	0.086716
Q9D1G3	Hhatl	-0.08742	0.510367	38.32737	37.30853	1.67E-04
Q14DK5	Hhipl1	-0.29567	0.388185	2.72113	3.64486	0.025978
Q99L13	Hibadh	0.16809	0.610951	24.40788	21.27586	0.3038

P43274	Hist1h1e	0.5076	0.251278	153.04325	140.48291	0.355361
P62806	Hist1h4a;	-0.1954	0.4157	99.51405	84.96115	0.013057
Q6PG16	Hjurp	1.9045	0.393851			
O08528	Hk2	0.81226	0.531236	204.13634	419.32049	0.021819
Q9D8V0	Hm13	-0.51774	NA			
P63158	Hmgb1	0.48034	0.479942	16.7975	20.94081	0.091247
Q8JZK9	Hmgcs1	-1.36969	0.140714	58.72093	391.59615	0.301165
Q01822	Hoxd1	-1.68706	0.388185	3.94585	4.87393	0.10909
Q6VYH9	Hsh2d	0.28537	0.345007	22.80467	17.77756	0.015952
P20029	Hspa5	-0.07587	0.639354	13.50514	14.36573	0.072973
P63017	Hspa8	0.36612	0.454112	5.11645	5.87245	0.078577
P38647	Hspa9	-0.02366	0.681669	7.52754	6.88777	0.15545
P63038	Hspd1	0.45167	7.94E-08	25.27962	24.28788	0.024621
Q64433	Hspe1	0.34752	0.007058	23.27505	21.03992	0.006809
P54071	Idh2	0.1197	0.321766	25.12987	22.32767	0.013263
Q9D6R2	Idh3a	0.32891	0.059572	23.17873	22.28914	0.019348
Q91VA7	Idh3b	0.17729	0.414697	12.46137	10.88742	0.194851
P70404	Idh3g	0.30559	0.031658	15.11195	14.12016	0.008064
Q64282	Ifit1	1.15642	0.139747			
Q8CAQ8	Immt	-0.22603	0.023959	29.84156	27.67556	0.006692
P85094	Isoc2a	0.25956	0.492804	11.73422	10.3759	0.17355
Q00651	Itga4	-0.1793	0.679532	303.02649	302.94285	0.09596
Q9JHI5	Ivd	0.26614	0.345007	16.78233	17.72977	0.114639
Q9ERI5	Jmjd6	0.78154	0.454112	17.58966	15.52824	0.009911
Q9ET80	Jph1	-0.09173	0.572045	10.04619	10.99991	0.000942
Q9ET78	Jph2	-0.55806	0.103832	14.88509	18.18844	0.025016
Q3MI48	Jsrp1	-0.01627	0.70504	13.47268	11.38646	0.28327

Q9D783	Kbtbd5	0.18472	0.661623	9.46771	9.15147	0.285469
P59111	Kcnh8	1.55322	0.245149			
Q61743	Kcnj11	0.37525	0.388185	319.52436	237.11953	0.187539
Q9DCV6	Kiaa0141	-0.36967	0.637095	25.85949	19.50589	0.217476
Q8BGX7	Kiaa0146	-0.55775	0.388185	0.90101	1.16984	0.001235
Q7TQE7	Kiaa0895	-0.24946	0.692918	38.79007	36.88511	0.264094
Q8K2Q9	Kiaa1598	0.04932	0.683928	9.29887	11.49613	0.125999
E9Q4K7	Kif13b	-0.37188	0.658714			
Q7TNC6	Kif26b	-0.34819	0.499299	4.83241	4.59139	0.244608
Q922S8	Kif2c	1.89767	NA			
Q61768	Kif5b	0.37075	0.500304	29.53107	26.57232	0.240911
Q8BUL5	Klhl7	-0.02809	0.70504	37.3191	33.09795	0.014708
P02535	Krt10	-0.01014	0.708326	74.70682	105.36121	0.000856
Q61782	Krt14	-0.97546	0.668743	40.56957	32.52522	0.059645
Q61781	Krt14	0.24418	0.637095			
Q9Z2K1	Krt16	0.03865	0.70504	108.99546	112.57859	0.025016
Q9QWL7	Krt17	-0.18511	0.567047	19.51953	18.44407	0.011018
Q6IFX2	Krt42	0.10242	0.668743	56.85824	54.24695	0.015642
Q922U2	Krt5	0.16017	0.668743	102.34969	93.66507	0.040781
P50446	Krt6a	0.14981	0.674733	132.11769	61.10444	0.348822
Q6IFZ6	Krt77	4.96523	0.458056			
Q8VED5	Krt79	-0.13163	0.668743	384.73409	356.09722	0.098503
Q9CXF0	Kynu	-1.50402	0.104449	9.39607	15.97681	0.178807
P11438	Lamp1	0.08386	0.674733	3.49571	2.88466	0.131336
Q9JKS4	Ldb3	0.05399	0.589517	23.17301	25.20854	0.154568
E9PYJ9	Ldb3	0.11488	0.687158	19.65905	20.2647	0.323994
P06151	Ldha	-0.84959	0.000131	9.88983	14.59446	0.007613

P16125	Ldhb	-1.71306	9.28E-08	18.95987	18.68382	0.197983
Q9Z2I0	Letm1	-0.09514	0.545905	13.93782	14.10459	0.034368
Q91VD1	Lgals12	-0.62461	0.373279	3.70327	5.02036	0.30583
Q8C129	Lnpep	-0.85867	0.007912	13.8218	1.72715	0.281399
Q505F5	Lrrc47	-0.6062	NA			
Q8K0B3	Lrrc66	3.37621	0.198146			
Q8K2F8	Lsm14a	0.62875	0.420405	17.55746	15.75345	0.01897
P97412	Lyst	0.16177	0.674733			
Q922B1	Macrod1	0.00672	0.709759	19.61303	19.72975	0.00491
Q9WTX8	Mad1l1	1.96064	0.674733			
Q8BW75	Maob	-0.5731	0.382454	8.22048	7.74373	0.012985
P27546	Map4	0.09523	0.687378	3.32555	4.29053	0.09919
P04247	Mb	-1.63768	7.44E-17	33.60481	26.94044	0.061077
P04370	Mbp	0.33239	0.392282	122.81639	79.41617	0.28327
Q3UMR5	Mcu	0.04265	0.679532	23.43354	21.23541	0.084972
P14152	Mdh1	-1.36486	1.39E-06	18.95943	21.80254	0.145722
P08249	Mdh2	0.39603	3.12E-07	30.47917	27.67406	0.230815
Q9DCS3	Mecr	0.56054	0.229447	33.6232	27.68373	0.114494
O88559	Men1	0.12615	0.692918	0.5191	0.2398	0.30583
Q811U4	Mfn1	-0.14817	0.674733	15.53027	13.22815	0.048642
A2AWL7	Mga	-0.05146	0.692918	6.9593	6.34301	0.072973
Q8BI84	Mia3	0.33777	0.236894			
Q8BMD7	Morc4	-0.13317	0.70504			
Q8VEL0	Mospd1	0.02402	0.709759	165.92463	71.24018	0.059123
P56379	Mp68	-0.15562	0.674733	27.51147	27.49112	0.03655
P63030	Mpc1	-0.32297	0.342238	9.69454	8.59891	0.041045
Q9D023	Mpc2	-0.15599	0.668743	10.77542	9.71521	0.040356

Q3TV65	Mpnd	-0.52672	0.405139			
Q99J99	Mpst	-0.28483	0.325187	6.80415	6.90034	0.305918
P27573	Mpz	-0.02668	0.708326	101.68134	108.81107	0.017244
Q3UR85	MRF	-3.78872	0.148096			
Q9CY16	Mrps28	0.36981	0.553469			
Q61733	Mrps31	-0.12439	0.666567	5.77288	4.85346	0.059204
Q9CQX8	Mrps36	0.28257	0.200093	27.0374	26.65535	0.051717
P03930	Mtatp8	-0.36441	0.154119	38.08575	35.12604	0.069504
Q791V5	Mtch2	-0.0408	0.668743	23.08903	22.73847	0.102515
P00397	Mtco1	-0.05391	0.668743	16.72383	15.40876	0.111112
P00405	Mtco2	0.01884	0.693113	25.76999	23.67003	0.244336
P00158	Mt-Cyb	-0.0154	0.711313	27.6715	25.67313	0.109633
Q9WTJ6	Mtl5	0.09038	0.661623	158.72546	99.23128	0.106378
P03888	Mtnd1	-0.02718	0.707709			
P03911	Mtnd4	-0.37968	0.479942	42.61332	39.47781	0.109633
P03921	Mtnd5	-0.18489	0.454112	23.11554	21.82689	6.69E-03
O88441	Mtx2	0.15992	0.454112	20.56199	16.01974	0.053689
Q5XKE0	Mybpc2	0.21714	0.3339	6.18067	4.54496	0.22774
P70402	Mybph	0.16969	0.65775	12.84946	12.094	0.072574
Q5SX40	Myh1	0.0687	0.5159	24.71914	27.92788	1.21E-10
B1AR69	Myh13	0.13549	0.665254	34.97686	35.78806	0.358064
P13541	Myh3	1.4115	0.136689	10.80144	13.7525	0.037267
Q5SX39	Myh4	0.51025	0.000296	16.87679	17.53711	6.69E-03
Q02566	Myh6	-0.23809	0.637095	4.11164	11.34335	0.040356
Q91Z83	Myh7	-0.06411	0.637095	28.35053	27.83775	0.005295
P13542	Myh8	-0.04548	0.668743	34.83029	34.56832	0.000623
P05977	Myl1	0.25262	0.154231	24.04147	28.94141	3.75E-05

P51667	Myl2	-0.4599	0.132107	30.81561	26.94172	0.248699
P09542	Myl3	-0.44141	0.083132	27.87636	25.91384	0.208346
Q8VCR8	Mylk2	-0.85744	0.603511	1906.03424	1143.76783	0.248655
P97457	Mylpf	0.2409	0.297889	32.28356	37.03824	1.52E-07
Q9JMH9	Myo18a	1.15487	0.342238	25.87108	5.10219	0.355361
Q62234	Myom1	0.215	0.207477	14.82125	15.94628	0.005775
Q9JIF9	Myot	0.19103	0.637095	10.30687	10.65925	0.28327
Q9JK37	Myoz1	-0.05812	0.674733	33.47807	43.46427	0.005867
Q8R4E4	Myoz3	-0.00554	NA			
Q8C339	NA	-3.19276	0.531236	53.46053	64.12323	0.135597
Q8BHE8	NA	-0.58892	0.687158	224.29168	98.79063	0.162103
Q8C5U4	NA	-0.54066	0.674733	16.02209	15.13844	0.20024
P06329	NA	-0.06519	0.583791	6.51256	6.46302	0.331908
Q6R5D8	NA	0.1317	0.668743			
Q3UNT5	NA	0.2838	0.708326			
Q8K2T4	NA	0.29863	NA			
Q9QUK4	Naip2	-0.08204	0.674733	16.91354	26.1956	0.17602
Q6GQX2	Nckap5l	-0.09895	0.674733	21.52498	18.7918	0.175935
O09000	Ncoa3	0.11568	0.611601	39.98473	33.24079	0.042738
Q9WU42	Ncor2	0.13625	0.674733	2.05168	1.57486	0.266484
Q9QYG0	Ndrg2	-1.29084	6.48E-05	3.75162	4.1493	0.154568
O35683	Ndufa1	-0.13332	0.583791	31.31408	32.98102	0.198747
Q99LC3	Ndufa10	-0.2758	0.08498	25.58647	22.64054	0.343003
Q9D8B4	Ndufa11	-0.66697	0.04742	19.28715	17.277	0.203476
Q7TMF3	Ndufa12	-0.35356	0.229447	17.31752	15.6864	0.060217
Q9ERS2	Ndufa13	-0.20628	0.245149	10.87152	10.50295	0.140905
Q9CQ75	Ndufa2	0.21233	0.392282	24.60902	22.76794	0.109633

Q9CQ91	Ndufa3	-0.13262	0.639354	19.91916	19.08703	0.175935
Q62425	Ndufa4	-0.1683	0.388185	13.25306	11.89862	0.031601
Q9CPP6	Ndufa5	-0.49782	0.089076	24.47284	23.10552	0.348822
Q9CQZ5	Ndufa6	-0.196	0.5364	13.76377	13.16876	0.263929
Q9Z1P6	Ndufa7	-0.17688	0.487973	12.9844	12.01368	0.026773
Q9DCJ5	Ndufa8	-0.19969	0.131523	29.25607	27.8894	0.010624
Q9DC69	Ndufa9	-0.10794	0.540127	23.29733	21.59101	0.070286
Q9CR21	Ndufab1	-0.15187	0.562852	36.12244	37.59764	0.177924
Q9DCS9	Ndufb10	-0.41129	0.008171	24.81814	22.73974	0.109633
O09111	Ndufb11	-0.2237	0.507525	19.45945	17.22366	0.061928
Q9CPU2	Ndufb2	-0.26106	0.445385	17.89612	15.45484	0.022294
Q9CQZ6	Ndufb3	-0.06031	0.633434	24.89553	22.74092	0.055958
Q9CQC7	Ndufb4	0.07851	0.57673	24.04744	22.77117	0.051816
Q9CQH3	Ndufb5	-0.06213	0.633434	26.55069	25.17669	0.198747
Q3UIU2	Ndufb6	-0.37524	0.329091	13.04255	13.16424	0.197273
Q9CR61	Ndufb7	-0.15114	0.502217	31.27583	30.63826	0.024664
Q9D6J5	Ndufb8	-0.22714	0.29108	28.04705	24.76758	0.084972
Q9CQJ8	Ndufb9	-0.20072	0.388185	30.19779	26.63706	0.184155
Q9CQ54	Ndufc2	-0.20688	0.236894	26.27527	25.8292	0.031218
Q91VD9	Ndufs1	-0.13014	0.149741	19.38702	18.17511	0.145903
Q91WD5	Ndufs2	-0.18983	0.080554	17.66332	17.12527	0.154568
Q9DCT2	Ndufs3	-0.13638	0.388185	19.91528	17.80112	0.02087
Q9CXZ1	Ndufs4	-0.05514	0.670041	15.3947	13.72705	0.244336
Q99LY9	Ndufs5	-0.21181	0.487973	25.86464	23.06215	0.210067
P52503	Ndufs6	-0.02471	0.668743	20.78609	18.87177	0.003628
Q9DC70	Ndufs7	-0.26897	0.433967	12.23846	11.87229	0.109633
Q8K3J1	Ndufs8	0.05111	0.668743	27.61826	22.65639	0.135947

Q91YT0	Ndufv1	-0.12562	0.11045	14.03612	13.21123	0.100767
Q9D6J6	Ndufv2	0.03751	0.661623	20.83007	18.89191	0.09596
Q8BK30	Ndufv3	-0.4388	0.345328	28.6233	27.91272	0.271887
A2AQB2	Neb	-0.53592	0.239203	21.97014	28.42189	0.064727
P08551	Nefl	-0.64046	0.350298	60.71865	101.25989	0.175557
P08553	Nefm	0.5654	0.275173	32.49224	26.72048	0.071317
Q9Z1J3	Nfs1	1.09824	0.610951	1.05569	4.49828	0.072973
Q01768	Nme2	-1.32169	0.022504	14.53411	20.0035	0.011011
Q9JKN6	Nova1	0.19694	0.668743	0.45063	0.72578	0.190136
Q60641	Nr1h4	0.55917	0.670985			
Q9DCN1	Nudt12	-0.45172	0.239203	19.82386	25.92841	0.039023
Q9DD16	Nudt22	-1.11095	0.098715	17.52609	14.7176	0.272549
A2AAJ9	Obscn	-0.15618	0.668743	16.60697	12.13657	0.040131
Q8CD62	Odz4	0.25238	0.690701			
Q60626	Ofa	-0.19542	0.700458			
Q60597	Ogdh	0.16561	0.092077	10.64154	9.59062	0.00751
P58281	Opa1	-0.51628	0.296886	11.3288	6.95552	0.269305
Q8CIV2	ORF61	0.49704	0.149741	5.32492	4.82529	0.016153
Q9D0K2	Oxct1	-0.36117	0.499299	16.08608	12.88172	0.060217
P09103	P4hb	-0.15536	0.245149	13.18993	11.04972	3.95E-02
Q99JB8	Pacsin3	0.38186	0.050552	5.20341	3.21431	0.205129
Q08642	Padi2	-0.29397	0.499299			
Q640Q5	Pan3	-0.03025	0.711313			
O88622	Parg	0.97118	0.149741	35.46172	18.13577	0.130417
Q99LX0	Park7	-1.19853	0.003906	41.07585	44.50456	0.106378
Q3TVI8	Pbxip1	-0.16375	0.559611	6.2343	5.5706	0.040356
Q05920	Pc	0.33169	0.236894	9.88735	11.55955	0.003013

P60335	Pcbp1	-1.56434	0.260014	2612.87368	607.57485	0.040356
Q99MN9	Pccb	0.35244	0.135314	39.4971	42.48308	0.189657
Q9QYX7	Pclo	-0.63079	0.577349			
P48725	Pcnt	0.10225	0.668743	23.83122	19.7516	0.025978
Q922S4	Pde2a	-1.95808	0.236894			
Q8CG03	Pde5a	0.06437	0.70504	17.80415	6.38221	0.279644
P35486	Pdha1	0.42855	4.09E-15	18.68391	17.11254	8.06E-02
P35487	Pdha2	-0.3001	0.545034	25.13546	17.57951	0.178807
Q9D051	Pdhb	0.28697	0.008171	38.32333	34.84688	0.356251
Q8BKZ9	Pdhx	0.32536	0.026499	37.69255	33.53397	0.134236
Q8CI51	Pdlim5	0.08015	0.668743	27.75525	30.13556	0.007549
Q3TJD7	Pdlim7	0.4403	0.467732	8.09537	8.58468	0.139851
P70296	Pebp1	-1.68451	6.65E-07	36.91544	46.05916	0.091561
P47857	Pfkm	-0.52057	0.000315	8.46517	7.86616	0.136976
O70250	Pgam2	-1.51233	1.88E-07	17.10383	26.70772	1.67E-04
P09411	Pgk1	-1.47892	2.05E-16	19.48252	25.38022	0.00369
Q9D0F9	Pgm1	-0.39976	0.143953	22.41033	28.54286	0.001996
P67778	Phb	-0.06834	0.477847	24.91597	22.52304	0.073549
O35129	Phb2	-0.05278	0.668743	29.26801	25.8968	0.038449
Q9DAK9	Phpt1	-0.62878	0.390619	8.98088	27.36097	0.12604
Q8BTP0	Pigz	0.05277	NA			
Q8K4R4	Pitpnc1	-4.403	0.435351			
P52480	Pkm	-0.87622	9.95E-17	17.693	18.31195	2.32E-05
P27612	Plaa	1.26869	0.510407			
P97813	Pld2	0.20835	0.674733	3.74367	2.78761	0.205363
Q9QXS1	Plec	-0.08638	0.668743	18.96092	17.76705	0.347003
Q9QZC7	Plekhb2	0.80372	0.245149			

O88492	Plin4	0.07205	0.670985	6.70292	6.37778	1.41E-02
J3QM92	Plscr5	0.18283	0.602239	6.1087	5.98319	0.135597
P17892	Pnliprp2	-0.11378	0.674733	10.16547	10.66137	0.1271
Q9D2C6	Polr3h	-1.67949	0.339292			
Q9DAC9	Pou5f2	-0.2227	0.611601	3.42176	3.4541	0.131766
Q3UM45	Ppp1r7	0.1928	0.447476			
Q61171	Prdx2	-1.29615	0.007912	21.83488	19.59139	0.212797
P20108	Prdx3	0.20442	0.414697	16.26893	15.95789	0.15557
P99029	Prdx5	-0.05589	0.662567	21.98708	18.86281	0.016343
Q9QUN5	Prl3c1	-0.68125	0.510986	25.25617	25.02421	0.08417
O55103	Prx	-0.04302	0.687158	74.74478	84.62563	0.006104
Q61207	Psap	0.24642	0.321766	3.66866	3.35415	0.177924
Q9JM51	Ptges	-1.28966	0.199813	23.24078	148.67756	0.017009
Q9QY80	Ptpla	0.05319	0.670985	138.04113	146.62819	0.159175
P32848	Pvalb	-1.08262	0.001258	31.55446	45.50959	0.000571
Q9ET01	Pygl	-0.33714	0.239203			
Q9WUB3	Pygm	-0.65521	7.48E-06	14.80279	14.07481	0.006692
P61027	Rab10	-0.06796	0.662567	6.11856	6.3322	0.216989
O35963	Rab33b	-0.18358	0.454112	5.16048	4.55825	0.101604
A2AWA9	Rabgap1	0.7166	0.492804	1.19652	0.76618	0.109396
P63001	Rac1	0.7231	0.376612	10.24212	18.08384	0.085728
Q75NR7	Recql4	-1.07465	0.430822	13.50537	20.90845	0.029989
Q9R1A8	Rfwd2	0.89698	0.479942			
Q9CQE5	Rgs10	-0.20433	0.665254	1.68511	2.82697	0.187539
Q4TU83	Rhox10	-1.46436	0.245149			
Q9CZM2	Rpl15	-0.14959	0.639354	8.39893	8.02701	0.249442
P62889	Rpl30	0.07898	0.674733	6.36093	6.77919	0.279439

P14869	Rplp0	-0.12523	0.692918	11.82705	9.04828	0.019499
Q91YQ5	Rpn1	0.0845	0.649698	31.33173	10.35246	0.025978
Q9WUT3	Rps6ka2	-0.01026	0.711313			
Q8CBB9	Rsad2	0.17601	0.658714	10.99939	0.56791	0.156788
O70622	Rtn2	-0.33076	0.000445	20.08962	19.16026	0.237903
E9PZQ0	Ryr1	-0.10744	0.136689	11.78843	11.50625	5.18E-06
Q8BGH2	Samm50	0.11401	0.477847	25.94118	23.26457	0.00441
Q8K2B3	Sdha	0.19965	0.000106	15.68347	14.37667	0.064727
Q9CQA3	Sdhb	0.01129	0.700139	11.69703	9.93099	0.188524
Q9CXV1	Sdhd	-0.17488	0.661174	113.12203	161.13421	0.15545
Q8R0F9	Sec14l4	-0.13247	0.668743	63.98506	54.60147	0.051678
P70274	Sepp1	-0.50732	0.367937	3.12438	9.11241	0.08417
O70456	Sfn	-0.26559	0.345007	327.79779	311.65334	0.320368
P82348	Sgcg	-0.58162	0.302474			
Q62141	Sin3b	0.36256	0.670242	50.05807	51.02578	0.091561
P46062	Sipa1	0.74544	0.445819	46.35972	68.19732	0.066873
Q8R4L0	Sla2	1.76306	NA			
Q9CR62	Slc25a11	-0.45534	0.500304	13.61131	6.61495	0.320913
Q8BH59	Slc25a12	-0.11624	0.248006	24.35844	22.53219	0.008027
Q9QXX4	Slc25a13	-0.16613	0.658983	31.57233	30.74684	0.159175
Q9Z2Z6	Slc25a20	-0.06447	0.668743	19.26321	17.57235	0.246463
Q8VEM8	Slc25a3	-0.31616	0.045125	21.56605	19.52875	0.266761
P48962	Slc25a4	0.11252	0.5364	31.33029	30.0153	0.197983
P51881	Slc25a5	0.33769	0.305453	15.72858	19.99909	0.001857
P14142	Slc2a4	0.14675	0.668743			
Q8BYF6	Slc5a8	-0.08891	0.676181			
Q9D8T7	Slirp	-1.70961	0.244479	7.69884	6.71242	0.323994

Q8C1Q6	SMIM4	-0.92323	0.507525	6.01039	6.7145	0.072973
Q8BQA2	Snap91	0.1495	0.661174	23.58468	16.72778	0.311722
P08228	Sod1	-1.03762	0.000445	21.58449	25.4832	0.084972
P09671	Sod2	0.05254	0.668743	29.89641	28.37999	0.150731
E1U8D0	Soga1	-0.29356	0.674733			
O88307	Sorl1	-0.23674	0.5159			
Q62407	Speg	-0.22511	0.665254	1.33856	7.98333	0.025016
Q3ULF4	Spg7	-0.92841	0.321766			
Q8C804	Spice1	-0.19105	0.690701	2769.10348	320.21056	0.058972
Q91WK1	Spryd4	-0.08177	0.637095	12.8019	10.84256	0.065013
Q7TQ48	Srl	-0.16775	0.245149	44.48882	43.81593	5.24E-07
Q9CYR0	Ssbp1	0.2526	0.499299	31.0884	20.99985	0.127806
Q76K27	St6gal2	-1.58369	0.236894			
Q8BZ71	Stac3	-0.68322	0.019501	1.2285	0.89238	0.188524
Q9JMD3	Stard10	0.41773	0.345007	2.58084	1.65519	0.090547
Q8C7E7	Stbd1	-0.38509	0.373279	3.98733	3.00653	0.223737
P83093	Stim2	-0.34776	0.668743	1.03375	4.26421	0.267116
Q99JB2	Stoml2	-0.94511	0.419278	24.95692	16.78182	0.106483
Q9Z2I9	Sucla2	0.21096	0.39841	17.8006	18.719	0.006914
Q9WUM5	Suclg1	0.42067	0.207044	31.63535	28.11522	0.192065
Q8BJS4	Sun2	-0.23809	0.668743	34.37043	16.31218	0.09919
Q6ZWR6	Syne1	0.39103	0.547663	5.36924	5.16499	0.323409
O89104	Sypl2	-0.06141	0.674733	68.92244	52.99109	0.061928
Q920N7	Syt12	-0.15624	0.637095	7.72492	4.5647	0.249442
P40749	Syt4	0.48984	0.198203	6.75779	7.04356	0.017005
Q7M724	Tas2r106	-0.49783	0.56736	79.57149	72.26717	0.137181
P70323	Tbx1	-0.53806	0.4327	6.15587	6.53279	0.088491

P23881	Tcea3	5.92994	0.388185			
P30051	Tead1	0.1686	0.668587	34.15658	42.42248	0.007522
Q8CD34	Tead3	-0.18329	0.547663	8.91932	5.38883	0.19654
Q9CY27	Tecr	0.21676	0.579513	95.99558	60.30134	0.084972
Q99MW5	Tex13	-1.13096	0.150956			
Q921I1	Tf	-0.20826	0.530876	1.74228	0.85988	0.296198
Q923W1	Tgs1	-0.22947	0.57673	1.06806	0.95511	0.198239
P63058	Thra	0.41632	0.043771	5.15941	4.24829	0.060217
Q69ZU6	Thsd7a	0.08416	0.674733	10.77648	6.84985	0.182929
P62075	Timm13	0.00374	0.711313	11.09827	11.36027	0.34338
Q9D880	Timm50	-0.39527	0.597102	15.89764	19.45929	0.051678
Q3UBX0	Tmem109	-0.16071	0.388185			
Q8C2L6	Tmem161b	-1.13222	0.584401	31.65371	63.03645	0.024664
Q9CZ16	Tmem178a	-0.32279	0.491446	20.61271	19.26437	0.095515
Q3TMP8	Tmem38a	-0.56839	0.388185	14.46633	11.48741	0.189657
P20801	Tnnc2	-0.06982	0.632945	15.76406	14.87152	0.156788
P13412	Tnni2	0.20406	0.40257	7.06907	8.25513	0.208346
Q9QZ47	Tnnt3	-0.09152	0.610976	11.43297	10.97219	0.069504
P17751	Tpi1	-0.80735	1.42E-06	22.56768	23.30341	0.000547
P58771	Tpm1	0.03704	0.670985	22.95236	22.78903	0.006692
P58774	Tpm2	0.12083	0.3339	19.77223	18.34935	0.011011
P21107	Tpm3	0.42872	0.479942	20.06608	14.40717	0.310892
Q1XH17	Trim72	-0.19515	0.051036	10.48567	10.71401	3.75E-05
Q9D0C4	Trmt5	1.10559	0.239134	5.37722	9.57635	0.051678
Q9Z2Q2	Tsg118	0.57069	0.430367	326.15779	292.81797	0.117683
A4Q9E4	Ttll2	0.18636	0.637095			
A2ASS6	Ttn	-0.17025	0.003338	22.57146	21.26374	2.36E-70

Q8BUJ0	Ttn	-0.11971	0.388185	20.70286	19.95559	4.46E-05
Q8BFR5	Tufm	0.35438	0.001525	24.04843	21.44635	0.051678
Q9ES34	Ube3b	-0.70288	0.477847	2.45705	7.07948	0.289437
P56501	Ucp3	0.29951	0.547663			
Q6P5E4	Uggt1	1.0771	0.616785	19.21337	18.27457	0.192801
Q91ZJ5	Ugp2	-1.18784	0.005749	19.03585	9.36761	0.080018
Q9D855	Uqcrb	-0.0388	0.668743	28.52417	28.14012	0.277024
Q9CZ13	Uqcrc1	-0.16937	0.070349	28.4586	27.22395	4.04E-02
Q9DB77	Uqcrc2	-0.20582	0.136689	29.41774	27.33841	0.298449
Q9CR68	Uqcrfs1	-0.20551	0.117718	20.80489	19.33859	0.000968
P99028	Uqcrh	-0.34488	0.417164	26.53048	25.83488	0.040289
Q9CQ69	Uqcrq	-0.62881	0.077786	29.09063	31.5736	0.152929
Q78IK2	Usmg5	-0.42908	0.160443	22.27072	18.21792	0.014906
P57080	Usp25	-0.59793	0.246071			
Q9QY76	Vapb	-0.19116	0.388185	30.291	25.83695	0.341121
Q01853	Vcp	0.56401	1.08E-05	7.14079	7.3645	1.67E-04
Q60932	Vdac1	-0.18819	0.08482	24.39965	22.81132	0.34338
Q60930	Vdac2	-0.10278	0.482594	22.49322	21.10303	0.293916
Q60931	Vdac3	-0.23579	0.160443	23.2367	20.71006	0.162926
Q9EQH3	Vps35	-0.21857	0.668743	17.63618	12.59618	0.205991
Q8R0J7	Vps37b	0.11153	0.637095	17.77669	16.6501	0.237712
Q4VBE8	Wdr18	0.37735	0.245149	29.16692	34.9384	0.012569
Q8BND3	Wdr35	-0.00497	0.711313	4.53411	2.00849	0.02146
Q9R0D8	Wdr54	-0.43749	0.510407	22.59312	16.43021	0.104198
Q9D565	Wdr64	0.5237	0.296886			
Q6P5F9	Xpo1	-0.52625	0.218467	5.94727	6.08699	0.075579
P46938	Yap1	-0.3722	0.668743	27.13824	2.98154	0.299048

P62259	Ywhae	-0.77441	0.010034	15.65966	14.47142	0.124826
P61982	Ywhag	-0.12698	0.668743	26.90062	19.80863	0.205363
Q69ZB8	Zcchc2	-0.45805	0.485869	729.73671	1116.34181	0.244336
P10755	Zfp14	-0.14151	0.632945	9.78395	7.06362	0.101347
Q9JLM4	Zmym3	-0.35761	0.584481	5.52297	6.81189	0.140387

Supplemental Table 3. Respiratory complex I changes of protein half-lives with age, q < 0.05.

	**EDL**			SOL		
Gene ID	**Old, $t_{1/2}$**	**Young, $t_{1/2}$**	**q value**	Old, $t_{1/2}$	Young, $t_{1/2}$	q value
ETF A	**27.72398**	**28.71458**	**4.20E-05**	25.85279	23.37246	1.55E-01
ETF B	**25.19793**	**25.19052**	**0.015724**	25.14121	23.5201	0.079401
ETF DH	**30.35514**	**30.13508**	**0.024708**	27.13726	25.8909	0.310997
ND4	**n.d.**			42.61332	39.47781	0.109633
ND5	**25.43842**	**22.19728**	**5.07E-05**	23.11554	21.82689	6.69E-03
NDUFA1	**32.29813**	**27.241**	**0.227699**	31.31408	32.98102	0.198747
NDUFA10	**26.68109**	**25.867**	**0.001577**	25.58647	22.64054	0.343003
NDUFA11	**22.00453**	**22.88779**	**0.054871**	19.28715	17.277	0.203476
NDUFA12	**18.40511**	**17.63694**	**0.066496**	17.31752	15.6864	0.060217
NDUFA13	**23.44089**	**19.41382**	**0.151624**	10.87152	10.50295	0.140905
NDUFA2	**27.50922**	**29.42165**	**0.089449**	24.60902	22.76794	0.109633
NDUFA3	**21.00021**	**19.76669**	**0.049385**	19.91916	19.08703	0.175935
NDUFA5	**25.28198**	**26.65287**	**0.008745**	24.47284	23.10552	0.348822
NDUFA6	**15.84313**	**15.35727**	**0.029718**	13.76377	13.16876	0.263929
NDUFA7	**12.12575**	**14.12613**	**0.003017**	12.9844	12.01368	0.026773
NDUFA8	**33.9086**	**31.01781**	**0.016579**	29.25607	27.8894	0.010624
NDUFA9	**23.85076**	**24.63906**	**0.00877**	23.29733	21.59101	0.070286
NDUFAB1	**41.91038**	**33.36359**	**0.213292**	36.12244	37.59764	0.177924
NDUFB10	**29.65731**	**28.04396**	**0.001657**	24.81814	22.73974	0.109633
NDUFB11	**24.94925**	**18.15107**	**0.174645**	19.45945	17.22366	0.061928
NDUFB2	**20.62173**	**18.15574**	**0.071883**	17.89612	15.45484	0.022294
NDUFB3	**29.9716**	**30.4589**	**0.093851**	24.89553	22.74092	0.055958
NDUFB4	**25.23159**	**23.48679**	**0.032283**	24.04744	22.77117	0.051816
NDUFB5	**30.93564**	**32.93646**	**0.142265**	26.55069	25.17669	0.198747
NDUFB6	**13.27428**	**3.36314**	**0.237063**	13.04255	13.16424	0.197273
NDUFB7	**38.12375**	**37.70128**	**0.020255**	31.27583	30.63826	0.024664
NDUFB8	**34.34346**	**30.35505**	**0.131738**	28.04705	24.76758	0.084972
NDUFB9	**33.3288**	**27.02252**	**0.114912**	30.19779	26.63706	0.184155
NDUFC2	**21.03532**	**18.32418**	**0.029254**	26.27527	25.8292	0.031218

NDUFS1	**20.1486**	**20.57246**	**0.00406**	19.38702	18.17511	0.145903
NDUFS2	**15.41848**	**15.11706**	**0.011837**	17.66332	17.12527	0.154568
NDUFS3	**19.08552**	**18.24347**	**0.026176**	19.91528	17.80112	0.02087
NDUFS4	**17.38605**	**15.9014**	**0.078292**	15.3947	13.72705	0.244336
NDUFS5	**27.07505**	**26.90405**	**0.020255**	25.86464	23.06215	0.210067
NDUFS6	**22.0896**	**20.84079**	**0.020255**	20.78609	18.87177	0.003628
NDUFS7	**11.16485**	**10.44628**	**0.059668**	12.23846	11.87229	0.109633
NDUFS8	**24.78909**	**20.97656**	**0.02829**	27.61826	22.65639	0.135947
NDUFV1	**17.3066**	**17.59706**	**0.00057**	14.03612	13.21123	0.100767
NDUFV2	**21.16207**	**18.78552**	**0.244024**	20.83007	18.89191	0.09596
NDUFV3	**33.60503**	**32.2185**	**0.136358**	28.6233	27.91272	0.271887
