# Supplementary material for: Age modifies respiratory complex I and protein homeostasis in a muscle type‐specific manner
Source: Aging Cell. 2015 Oct 25;15(1):89–99. doi: 10.1111/acel.12412 (PMC4717270; doi:10.1111/acel.12412)
Supplement: Supplementary file 2 [file ACEL-15-089-s002.docx]

**Figure 1. Mitochondrial Protein Half-life is Modified by Age and is Dependent on Muscle Type**

(A) Plots showing significant changes in mitochondrial protein half-life with age. Changes were roughly half increasing and half decreasing in half-life in EDL, while the majority of mitochondrial proteins in SOL increased half-life (slower turnover rate). Proteins with half-life longer than one year excluded from analyses. (B) Venn diagrams showing few mitochondrial proteins decreased half-life in aged SOL, whereas those that increased half-life were similar to those that increased half-life with age in EDL. q value < 0.05.

**Figure 2.** **Protein Abundance is Modified by Age and Muscle Type (A)** Protein abundance was altered with age and the largest change was in EDL; 137 proteins significantly (adjusted p value [q value] < 0.05) increased in content with age in EDL among 745 total proteins detected. 108 of these proteins were mitochondrial, comprising over 96% of mitochondrial proteins that changed with age in the EDL. **(B)** Comparing citrate synthase activity between mitochondrial enriched fractions and whole cell homogenate shows that the efficiency of extraction was not different in young vs. aged tissues and was not different between EDL and SOL in either age group. **(C)** Measurement of the AUC of all mitochondrial proteins identified using on an online database (mitoP2, <http://www.hsls.pitt.edu/obrc/index.php?page=URL1097158105>) relative to total AUC indicates that mitochondrial content of aged EDL is increased relative to young EDL. **(D)** The direction of change with age was often different between EDL and SOL. Heat map of old/young ratio of protein abundance grouped by Ingenuity Pathway Analyses (IPA) canonical pathways. Top three pathways shown, ordered left to right by the significance of the pathway change with age, q <0.05 increased with age in darker red, and decreased with age in darker blue. Some proteins occur in more than one pathway. EDL top, SOL bottom rows. IPA of Proteins that change abundance with age, q < 0.05, excluding pathways with less than 4 gene products that changed with age, listed in Supplemental Table 2.

**Figure 3.** **Age Causes Changes in Respiratory Capacity and Mitochondrial Content**

Respiration in fast twitch EDL **(A)** was maintained with age, but decreased in slow twitch SOL **(B)**, n = 12 ─24. Mitochondrial respiratory protein content of representative subunits increased with age in EDL **(C)** while stable or decreased in SOL **(D)**, n = 10 ─17. Inset shows western analyses of respiratory components CI subunit NDUFB8, CII 30kDa subunit, and CIV subunit I. 42 kD Ponceau (actin) band was used to normalize protein load. Data are expressed relative to young EDL or SOL. Respiratory flux per mitochondrial content is expressed as a fraction of the young control value **(E and F)**, n = 8. *p < 0.05, **p < 0.01, ***p < 0.001.

**Figure 4.** **Effect of Age on Expression and Half-life of ETS Components in the EDL**

Map of complex I respiratory apparatus, electron transfer flavin (ETF) moiety and table of other respiratory complexes (mitochondria-encoded in bold) showing changes in protein abundance (Ab) and half-life (t_1/2_). *NDUFA9, traditionally associated with complex I, may be associated instead with complex IV (Balsa et al. 2012). n.d. = not detected.

**Figure 5.** **Improved Age Related Dysfunction of Complex I Respiratory Capacity, Efficiency and Protein Content in mCAT Mice**

Respiration in aged mCAT mice **(A)** was elevated in EDL and **(B)** not significantly changed in SOL compared to WT, n = 5 ─12. **(C)** Complex I content of aged EDL in mCAT mice is decreased compared to WT aged mice (aged from Figure 3C). Inset shows western analyses of respiratory components CI subunit NDUFB8. 42 kD Ponceau (actin) band was used to normalize protein load. Shown relative to young EDL or SOL, n = 11. **(D)** Complex I respiratory flux per mitochondrial content of NdufB8 is expressed as a fraction of the young control value (aged from Figure 3D). All data sets in figure D were significantly different than hypothetical mean of 1 (young) with the exception of aged mCAT EDL, which was also significantly different than aged mice (T-test), n = 8. *p < 0.05, **p < 0.01.

**Figure 6. Schematic Model of Mitochondrial Dysfunction with Age**

Mitochondrial protein quality control declines in susceptible tissue while oxidative stress increases with age. This leads to mitochondrial dysfunction presented by accumulation of mitochondrial respiratory proteins to compensate for decrease mitochondrial quality. Mitochondrial-targeted antioxidant (mCAT) improves protein quality with age resulting in improved mitochondrial function.

**Supplementary Figure 1**. **Protein Half-life is Modified with Age and is Dependent on Muscle Type (B)** Heatmap showing changes in protein half-life. Mitochondrial proteins are grouped by IPA canonical pathways and ordered left to right by the significance of the pathway half-life change with age, q value < 0.05.

**Supplemental Figure 2. Minimal Inverse Correlation between Protein Abundance and Half-life**

EDL Y = -0.1586*X + 0.04215, r^2^ = 0.01907, slope significantly non-zero p = 0.0018.

SOL Y = -0.1720*X + 0.1376, r^2^ = 0.03374, slope significantly non-zero p = < 0.0001.

**Supplemental Figure 3. EDL and SOL Tissue Weights**

Skeletal Muscle was decreased in weight with age in both EDL and SOL. **(A)** Relative to body weight, n = 20. **(B)** relative to tibialis anterioris length, n = 9, *p < 0.05.

**Supplementary Figure 4. Oxidative State of Skeletal Muscle: Muscle Fiber H_2_O_2_ Production, Protein Carbonyl content and GSH-modified Proteins**

**(A)** Mitochondrial H_2_O_2_ production in permeabilized muscles was elevated with age in both muscles, n = 8 ─11. **(B)** H_2_O_2_ production with age relative to VDAC1 (mitochondrial content) was significantly higher in only the EDL, n = 5 ─10. **(C)** However, protein damage as measured by protein carbonyl content did not change with age in either muscle, EDL n = 7, data normalized to young EDL. **(D)** Protein glutathionylation increased with age in only the SOL, n = 9. *p < 0.05, ***p < 0.001.
